# Effectiveness of mHealth App–Based Interventions for Increasing Physical Activity and Improving Physical Fitness in Children and Adolescents: Systematic Review and Meta-Analysis

**DOI:** 10.2196/51478

**Published:** 2024-04-30

**Authors:** Jun-Wei Wang, Zhicheng Zhu, Zhang Shuling, Jia Fan, Yu Jin, Zhan-Le Gao, Wan-Di Chen, Xue Li

**Affiliations:** 1 School of Sport Medicine and Health Chengdu Sport University Chengdu China; 2 School of Sports Science Beijing Sport University Beijing China; 3 Physical education institute Xinyu University Xinyu China; 4 Academic Administration Chengdu Sport University Chengdu China

**Keywords:** mobile health, mHealth apps, children and adolescents, physical activity, physical fitness, systematic review, meta-analysis, mobile phone

## Abstract

**Background:**

The COVID-19 pandemic has significantly reduced physical activity (PA) levels and increased sedentary behavior (SB), which can lead to worsening physical fitness (PF). Children and adolescents may benefit from mobile health (mHealth) apps to increase PA and improve PF. However, the effectiveness of mHealth app–based interventions and potential moderators in this population are not yet fully understood.

**Objective:**

This study aims to review and analyze the effectiveness of mHealth app–based interventions in promoting PA and improving PF and identify potential moderators of the efficacy of mHealth app–based interventions in children and adolescents.

**Methods:**

We searched for randomized controlled trials (RCTs) published in the PubMed, Web of Science, EBSCO, and Cochrane Library databases until December 25, 2023, to conduct this meta-analysis. We included articles with intervention groups that investigated the effects of mHealth-based apps on PA and PF among children and adolescents. Due to high heterogeneity, a meta-analysis was conducted using a random effects model. The Cochrane Risk of Bias Assessment Tool was used to evaluate the risk of bias. Subgroup analysis and meta-regression analyses were performed to identify potential influences impacting effect sizes.

**Results:**

We included 28 RCTs with a total of 5643 participants. In general, the risk of bias of included studies was low. Our findings showed that mHealth app–based interventions significantly increased total PA (TPA; standardized mean difference [SMD] 0.29, 95% CI 0.13-0.45; *P*<.001), reduced SB (SMD –0.97, 95% CI –1.67 to –0.28; *P*=.006) and BMI (weighted mean difference –0.31 kg/m^2^, 95% CI –0.60 to –0.01 kg/m^2^; *P*=.12), and improved muscle strength (SMD 1.97, 95% CI 0.09-3.86; *P*=.04) and agility (SMD –0.35, 95% CI –0.61 to –0.10; *P*=.006). However, mHealth app–based interventions insignificantly affected moderate to vigorous PA (MVPA; SMD 0.11, 95% CI –0.04 to 0.25; *P*<.001), waist circumference (weighted mean difference 0.38 cm, 95% CI –1.28 to 2.04 cm; *P*=.65), muscular power (SMD 0.01, 95% CI –0.08 to 0.10; *P*=.81), cardiorespiratory fitness (SMD –0.20, 95% CI –0.45 to 0.05; *P*=.11), muscular endurance (SMD 0.47, 95% CI –0.08 to 1.02; *P*=.10), and flexibility (SMD 0.09, 95% CI –0.23 to 0.41; *P*=.58). Subgroup analyses and meta-regression showed that intervention duration was associated with TPA and MVPA, and age and types of intervention was associated with BMI.

**Conclusions:**

Our meta-analysis suggests that mHealth app–based interventions may yield small-to-large beneficial effects on TPA, SB, BMI, agility, and muscle strength in children and adolescents. Furthermore, age and intervention duration may correlate with the higher effectiveness of mHealth app–based interventions. However, due to the limited number and quality of included studies, the aforementioned conclusions require validation through additional high-quality research.

**Trial Registration:**

PROSPERO CRD42023426532; https://tinyurl.com/25jm4kmf

## Introduction

### Background

The COVID-19 global pandemic has had adverse effects on the physical fitness (PF) and mental health of children and adolescents [1]. Before the COVID-19 pandemic outbreak, only approximately 30% of children and adolescents worldwide could meet the recommended levels of physical activity (PA) [2,3]. However, the COVID-19 pandemic has exacerbated this issue by decreasing their levels of PA, increasing sedentary behavior (SB), and leading to a decline in their PF [4]. A recent study in the United Kingdom discovered that children exhibited lower performance scores on the seated forward bend and 20-meter shuttle run test and higher BMI values in 2020 than in 2019 [5]. PF is a crucial determinant of children’s and adolescents’ health status [6], which can be influenced by various factors, including genetic, environmental, and PA-related factors [7]. Epidemiological studies have established a “dose‒response” relationship between PA and PF, which showed that increased PA levels and reduced SB were positively associated with improved PF in adolescents [8]. Therefore, the way to increase PA and improve PF is still one of the most important social problems to be solved for children and adolescents.

The rapid advancement of intelligent technology has increased the use of smartphones among young generation and the wide use of mobile health (mHealth) technologies [9,10]. At present, mHealth technologies, including wearable devices, smartphones, tablets, mHealth apps, smartwatches, and pedometers, are commonly used in the field of health care [11]. Recently, 2 systematic reviews have investigated the impact of mHealth-based interventions on behavioral changes, including PA and SB, in children and adolescents [12,13]. However, these reviews primarily concentrated on specific mHealth technology interventions, including SMS text messaging, wearable devices, web-based interventions, and others. Moreover, these systematic reviews exclusively focus on one or more behavioral changes, encompassing physical inactivity and SB, and the overall quality of the evidence is deemed low [13]. Current research indicates that app-based interventions on smartphones may represent the most effective strategy [13]. mHealth app–based interventions are among the most commonly used methods within the realm of mHealth technologies. Among these technologies, mHealth apps have been extensively used in the fitness and medical fields due to their affordability, personalization, and diverse features [14]. mHealth apps can provide quantitative visual feedback regarding the health behaviors of users, such as their PA; meanwhile, users upload their personal information to app databases, and apps facilitate personalized, long-distance, and low-contact training to improve the healthy development of users [15]. In recent years, mHealth app–based interventions have shown significant promise in promoting healthy behaviors, including increased PA and reduced SB, among children and adolescents. Nevertheless, there is a lack of systematic reviews comprehensively summarizing the impacts of stand-alone mHealth apps or concerted interventions using apps as one of the multiple components (eg, behavioral counseling combined with app interventions) on various health behaviors, including PF. In addition, studies in this domain have been predominantly centered on adults, with a noticeable dearth of pertinent research within populations such as children and adolescents [16].

Studies have demonstrated that mHealth app–based interventions can lead to effective outcomes in improving the PA behavior of users [17]. However, another study [18] found that such intervention has indicated only small effects on PA and is likely related to potential influencing factors. Furthermore, the efficacy of mHealth app–based interventions on PF is inconsistent. One study linked lower BMI and higher motor competence to the frequency and type of mHealth app use [19], while another study indicated that mHealth app–based interventions were ineffective in improving PF among adolescents, which is possibly due to the characteristics of the intervention [20]. The use of theory-based mHealth app interventions may also be more advantageous in increasing PA and enhancing PF in children and adolescents [21]. Several theoretical paradigms, including self-determination theory (SDT), the transtheoretical model, the health belief model, the theory of planned behavior, and social cognitive theory (SCT), have been used in mHealth app–based interventions [22]. The number and type of behavior change technique (BCT) clusters may also play a significant role in the effectiveness of mHealth app interventions. Michie et al [23] provided a standardized taxonomy of BCT that categorizes them into 16 clusters, such as feedback and monitoring, reward and threat, goals and planning, shaping knowledge, social support, and comparison of outcomes. This taxonomy aids in identifying which BCT clusters are more effectively applied to apps, thereby enhancing PA promotion and PF improvement. In conclusion, various factors, including the type of mHealth app, intervention characteristics, theoretical paradigms, and BCT clusters, are important considerations in the effectiveness of mHealth app interventions. Despite the increasing number of articles summarizing interventions based on mHealth apps, a noteworthy research gap persists. Most of these articles concentrate on interventions using commercially available mHealth apps that lack evidence-based behavior change strategies. Nevertheless, a significant proportion of users and patients rely on commercially available app**-**based mHealth interventions that lack empirical evaluation and rarely incorporate evidence-based behavior change strategies. Furthermore, current research predominantly highlights the intervention effects of mHealth apps on health-related outcome measures. However, there is a notable deficiency in evidence-based mHealth apps intervention programs and studies that integrate various target behaviors.

### Objectives

The first objective of this systematic review aims to evaluate the effectiveness of mHealth app–based interventions in promoting PA and improving PF among children and adolescents. The second objective is to specifically assess moderating effects (eg, age, types of apps, theoretical paradigm, BCT clusters, and intervention duration) on the effectiveness of mHealth app–based interventions in subgroups within these studies. Unlike previous studies, our review contributes evidence-based, high-quality content for potential mHealth app interventions addressing PA and PF. This contribution results from a meticulous evaluation and meta-analysis of relevant randomized controlled trials (RCTs). In addition, we conducted an extensive analysis of key moderating variables using subgroups and meta-regression, encompassing theoretical paradigms, BCT clusters, intervention duration, and more. This comprehensive approach enhances our understanding of the factors influencing intervention effects and facilitates the precise quantification of the intervention program. Our endeavors significantly expand the research scope beyond previous reviews.

## Methods

### Registration and Approval

The systematic reviews were registered on the PROSPERO (CRD42023426532). The literature search, reporting guidance, and implementation process of the study followed the PRISMA (Preferred Reporting Items for Systematic Reviews and Meta-Analyses) guidelines [24].

### Search Strategy

Several databases were searched, including PubMed, Web of Science, EBSCO, and Cochrane Library, to identify relevant RCTs published until December 25, 2023. The search strategy involved a Boolean search using a combination of subject-related words and free words. The following search terms were used: (Child OR Preschool OR Adolescent), (“Mobile health application*” OR “mHealth app*” OR “Portable Software Application*” OR “Mobile Application*” OR App*), and (“Physical Activity” OR PA OR MVPA OR “sedentary behavior” OR SB OR “Physical Fitness”). A detailed search strategy for Web of Science is presented in Multimedia Appendix 1. We opted to update the searches in the same databases used for the initial search to refine the results for this study. In addition, we examined the references cited in previous reviews to identify additional relevant literature. Concurrently, we reached out to authors of potentially eligible studies to obtain complete data. If >2 attempts were made to contact authors without receiving a response, the study was excluded. The literature search was not restricted by language.

### Inclusion and Exclusion Criteria

#### Inclusion Criteria

The following criteria were included for inclusion in the literature review: (1) The study comprised children and adolescents, aged 3 to 18 years, with the majority falling within this range. Participants did not exhibit physical dysfunction, and overweight or obesity, among other factors, were not exclusionary criteria. Children and adolescents were categorized into 3 groups: preschoolers aged 3-6 years; children aged 7-12 years; and adolescents aged 13-18 years. (2) Interventions using smartphone-based and tablet-based mHealth apps may involve either stand-alone apps (ie, solely apps) or concerted intervention (ie, apps combined with another intervention). The control group comprised genuine controls, such as no interventions, waitlist conditions, and usual clinical care. In addition, active controls, including interventions via apps, were considered. Studies using placebo and sham apps also met the inclusion criteria. (3) The study design was an RCT. (4) Primary outcome measures included PA, SB, performance-related PF (eg, coordination and balance), health-related PF (eg, cardiorespiratory endurance, muscle strength, and body composition), and physiological function (eg, body shape and metabolism).

#### Exclusion Criteria

The exclusion criteria were as follows: (1) articles written in languages other than English and Chinese; (2) repeated published studies, basic studies, observational studies, reviews, and case series articles; (3) studies for which full text was unavailable or data were incomplete; and (4) mHealth apps that only used SMS text messaging interventions or were incompatible with smartphones or tablets.

### Study Selection

After the literature search, the initial search results were imported into EndNote 20 (Thomson ResearchSoft) to remove duplicate articles. Predefined inclusion and exclusion criteria were applied to the literature. Two authors (ZZ and ZS) initially screened the titles and abstracts. Articles meeting the criteria were downloaded, and 1 author (ZZ) thoroughly evaluated the full text based on the inclusion and exclusion criteria, while the other author (ZS) conducted a randomized assessment. Another author (JF) was involved in resolving discrepancies between 2 independent reviewers to determine if a study met the inclusion criteria.

### Data Extraction

The included literature was independently extracted by 2 researchers (YJ and ZLG). The extracted information included basic details (eg, authors, publication year, country, and study type), characteristics of the study population (eg, age, gender, and sample size), characteristics of mHealth apps (eg, name, type, theoretical paradigm, and number or type of BCT clusters), intervention characteristics, outcome measures, and indicators related to risk of bias assessment. Two authors (YJ and ZLG) assessed the use of BCT in apps using the taxonomy by Michie [23]. Relevant information was primarily extracted from the study descriptions; in cases of incomplete data, the original apps were consulted. Disputes were resolved through third-party consultation (WDC).

### Risk of Bias Assessment

Two independent (YJ and ZLG) investigators assessed the risk of bias using the Cochrane Working Group’s tool [25]. Any disagreements were resolved by a third independent researcher (WDC) through consultation. Each study underwent evaluation in 7 domains, and the risk of bias was categorized as unclear, low, or high.

### Statistical Analysis

If the number of included papers was <3, then a systematic review was conducted. When a sufficient number of included studies were available for a meta-analysis, we used Revman 5.4 (Nordic Cochrane Center) and Stata 16.0 (StataCorp) to estimate effect sizes, conduct subgroup analysis, and sensitivity analysis. Weighted mean difference (WMD) and 95% CI were used as effect measures when the same measurement method was used. When measurement methods were consistent, the standardized mean difference (SMD) and 95% CI were used. The magnitude of SMD was interpreted as follows: SMD<0.2, negligible; 0.2≤SMD<0.5, small; 0.5≤SMD<0.8, medium; and SMD≥0.8, large [26].

The study indirectly mentioned the mean and SD, and the MD and SMD were calculated as the postintervention mean and SD based on the Cochrane Handbook [27]. The study examined the magnitude of heterogeneity using *I*^2^ and *P* values. If *I*^2^≤50% and *P*≥.10, a fixed-effect model was used for data analysis. On the contrary, if *I*^2^>50% and *P*<.10, a random effect model was used for meta-analysis [27]. The sources of heterogeneity were identified by subgroup analyses and meta-regression analysis based on type of apps, theoretical paradigm, age, and BCT clusters. The robustness of each study was evaluated using sensitivity analysis, and publication bias was assessed with funnel plots and the Egger test. Results

### Study Selection

**Figure 1:**
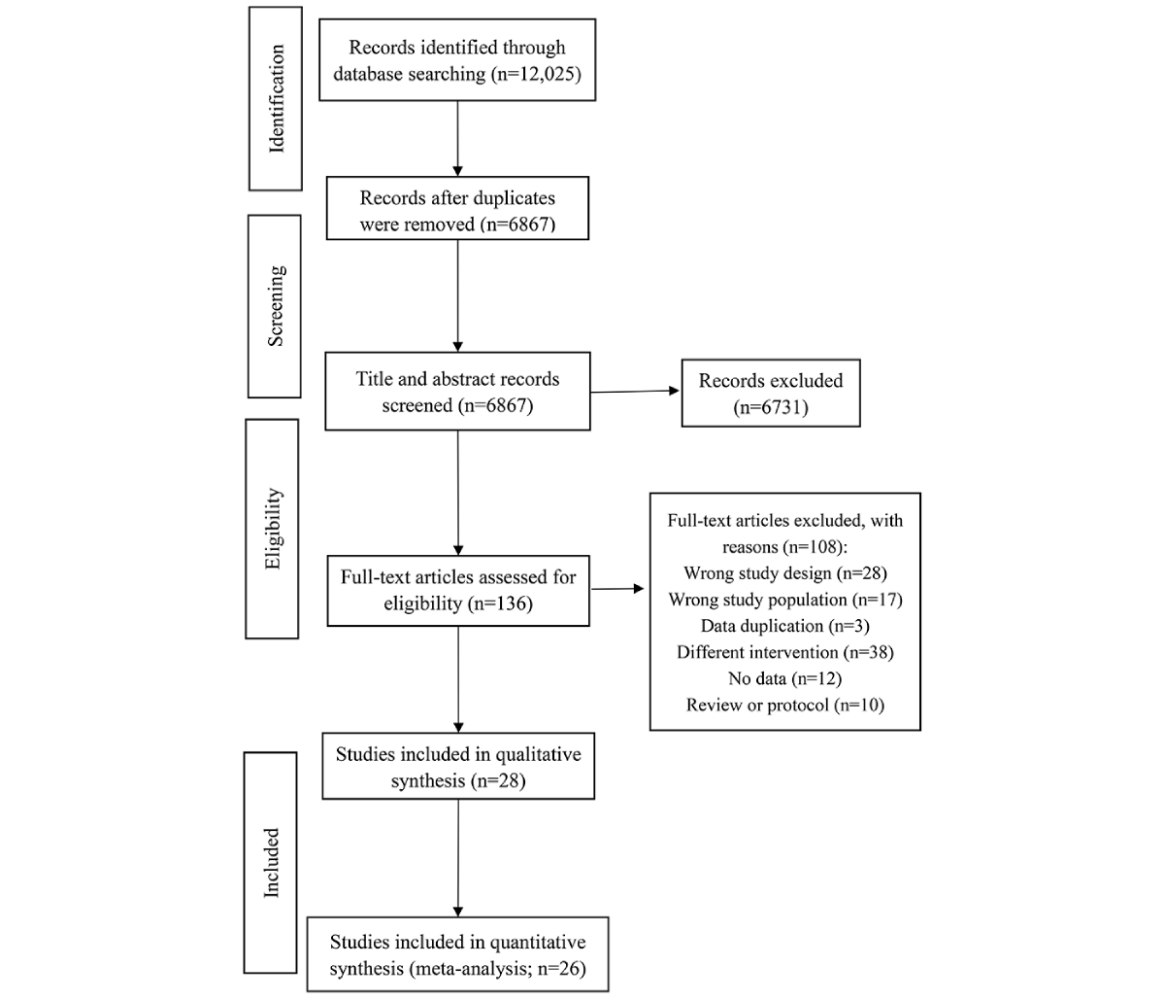
Flowchart of the study selection.

A total of 12,025 relevant articles were retrieved from PubMed (n=69), Web of Science (n=11,033), EBSCO (n=217), and Cochrane Library (n=706). Duplicate references were removed, which resulted in 6867 articles. Following an initial screening by abstracts, 136 articles were identified, which were then assessed by reading the full text. Finally, 28 articles were chosen for inclusion (Figure 1).

### Characteristics of the Included Studies

This study included 28 publications, all of which were RCTs published between 2014 and 2023 [20,26,28-49]. The sample size included 5643 subjects, ranging from 12 to 1392 participants per study. As shown in Table 1, the included studies have the following basic characteristics. The age of the participants varied from 3 to 18 years with 5 (18%) of the 28 studies involving preschool children [26,44,46,48,50], 8 (29%) studies involving children [28-31,33,36,37,51], and 15 (53%) studies involving adolescents [20,32,34,35,38-43,45,47,49,52,53]. The objectives of the studies differed, and the selection criteria for the target population varied inconsistently. Most studies (16/28, 57%) focused on healthy children and adolescents; however, of the 28 studies, 9 (32%) included participants with overweight and those with obesity [29-31,35,36,39,43,45,52], 2 (7%) involved children with cancer [34,53], and 1 (4%) enrolled youth with congenital heart disease [40]. Of the 28 studies, 8 (29%) were conducted in Asia [31,36,37,39-41,44,45], while the remaining studies were performed in Oceania (7/28, 25%) [29,30,35,38,46,50,53], North America (5/28, 18%) [28,33,34,43,51], and Europe (8/28, 29%) [20,26,32,42,47-49,52].

This review encompassed 28 studies that used 14 different mHealth apps. These included 9 commercial apps [20,31-33,35,38,41,47,49] and 14 research apps [28-30,39,40,42-46,48,51-53], and 5 mHealth apps did not provide the corresponding information [26,34,36,37,50]. The type of intervention used in 15 studies was stand-alone apps [20,26,28,32,33,40,41,43-47,49-51], and 13 studies used concerted intervention [29-31,34-39,42,48,52,53]. Participant engagement with the intervention was mentioned in 28 studies, 8 studies focused on parent-centered [26,31,36,37,44,46,48,50], the remaining studies were child centered. The mHealth apps were based on various theoretical paradigms, including self-regulation theory (SRT), SDT, and SCT. Different numbers or types of BCT clusters were identified, and they ranged from 1 to 7 clusters. Examples of BCT clusters used included goal setting and planning, feedback and monitoring, and behavioral comparison. Interventions duration lasted 2 to 48 weeks (Table 1). The primary and secondary outcome measures included total PA (TPA), moderate to vigorous PA (MVPA), SB, cardiorespiratory fitness (CRF), BMI, waist circumference (WC), muscle strength, agility, flexibility, muscular power, and endurance.

**Table 1:** Summary of the intervention characteristics of the included studies.

Study	Participants or population	Age (years)	Sample size （treatment/control）	Interventions	Comparator	Type of mobile health apps	Theoretical paradigm	BCT^a^ clusters	Duration（weeks）	Outcomes
Direito et al [20]	Insufficiently active healthy young people	15.7 (+1.2 or –1.2)	32/17	Group 1: immersive app *Zombies Run;* group 2: nonimmersive app *Get Running*	No interventions	Commercial apps	SRT^b^	Feedback and monitoring	8	CRF^c^, TPA^d^, SB^e^, and MVPA^f^
Garde et al [28]	Healthy students	11.3 (+1.2 or –1.2)	26/16	MKMM game app	Waitlist (crossed over after 3 weeks)	Research apps	SDT^g^ and theory of motivation	Reward and threat feedback and monitoring	4	TPA
Lubans et al [29]	Adolescent boys “at risk” of obesity	12.7 (+0.5 or –0.5)	181/180	Face-to-face PA^h^ sessions+pedometers for PA self-monitoring+purpose-built web-based smartphone apps+other	Regular curriculum	Research apps	SCT^i^ and SDT	Goals and planning, shaping knowledge, social support, and feedback and monitoring	32	SB, MVPA, BMI, WC^j^, muscle strength, and muscular endurance
Smith et al [30]	Adolescent boys “at risk” of obesity	12.7 (+0.5 or –0.5)	181/180	Face-to-face PA sessions+pedometers for PA self-monitoring+purpose-built web-based smartphone apps+other	Regular curriculum	Research apps	SCT and SDT	Goals and planning, shaping knowledge, social support, feedback and monitoring	20	TPA, SB, MVPA, BMI, WC, and muscle strength
Fernandez-Luque et al [31]	Children with overweight and those with obesity	9-12	108/119	Wearable sensors+mobile and social media (WhatsApp and Instagram)	No intervention	Commercial apps	NR^k^	Goals and planning, social support, feedback and monitoring	12	BMI
Pyky et al [32]	Young adolescent men	17.8 (+0.6 or –0.6)	250/246	*MOPOrtal* app	No intervention	Commercial apps	TTM^l^	Goals and planning, shaping knowledge, feedback and monitoring, and comparison of outcomes	24	SB and BMI
Gaudetet al [33]	Young adolescents	13 (+0.35 or –0.35)	23/23	An individualized goal was set by *Fitbit app*	No intervention	Commercial apps	SRT and self-monitoring theory	Goals and planning, feedback and monitoring, and regulation	7	MVPA
Mendoza et al [34]	Adolescent and young adult childhood cancer survivors	16.6 (+1.5 or –1.5)	29/20	Fitbit Flex wearable wristband and mobile health app+peer-based web-based support group	Usual care	NR	SDT	Goals and planning, feedback and monitoring	0	SB and MVPA
Nyström et al [26]	Healthy Swedish children	4.5 (+0.1 or –0.1)	156/159	MINISTOP app	Information or advice about a healthy diet+PA via a 4-page pamphlet	Research apps	SCT	Shaping knowledge, feedback, and monitoring	24	SB and MVPA
Chen et al [35]	Adolescents who are overweight or obese	14.9 (+1.67 or –1.67)	23/17	Fitbit Flex app+iStart Smart for Teens web-based educational program+biweekly SMS text messages	Omron HJ-105 pedometer+a blank food and activity diary	Commercial apps	SCT	Goals and planning, feedback and monitoring, shaping knowledge, social support, regulation, natural consequences, and covert learning	12.24	TPA, SB, and BMI
Browne et al 2020 [52]	Children with obesity	9-16	8/12	Usual clinical care+Mandolean training (*myBigO app*)	Usual clinical care	Commercial apps	NR	Goals and planning, feedback and monitoring, comparison of outcomes, shaping knowledge, social support, repetition and substitution, and antecedents	4	BMI
Garde et al [51]	Elementary school students	10.6 (+0.51 or –0.51)	19/18	*MKMM* game app	No intervention	Research apps	SDT and theory of motivation	Goals and planning, feedback and monitoring, comparison of outcomes, and social support	2	TPA
Trost and Brookes [50]	Children	3-6	17/17	*Moovosity* app	No intervention	NR	NR	NR	8	TPA
Devine et al [53]	Adolescent and young adult survivors of childhood cancer	13-25	25/24	In-person group sessions+mobile app+fitness tracker use alone	Waitlist	Research apps	SCT	Goals and planning, feedback and monitoring, shaping knowledge, and social support	12	CRF, SB, MVPA, BMI, WC, muscle strength, and coordination
Kahana et al [36]	Children with overweight and obesity	Median 10	32/47	Structured PA sessions, nutritional and behavioral counseling+“Just Dance Now” and “Motion Sports” app	Structured PA sessions, nutritional, and behavioral counseling	NRNR	NR	—^m^	20	BMI, muscle strength, muscular power, muscular endurance, and agility
Liu et al [37,54]	Primary school children	9.6 (+0.4 or –0.4)	705/687	Health education reinforcement of PA and BMI monitoring and feedback (*Eat Wisely and Move Happily* app)	Health education lessons and physical education sessions	NR	NR	Feedback and monitoring	36	CRF, TPA, MVPA, BMI, WC, muscular power, and muscular endurance
Likhitweerawong et al [39]	Children and adolescents with obesity	10-15	35/35	OBEST app+standard care	Standard care	Research apps	Theory of motivation	Goals and planning, feedback and monitoring, and shaping knowledge	24	BMI and WC
Lin et al [40]	Youth with congenital heart disease	15-24	100/50	Group 1: *COOL Passport* app; group 2: *COOL Passport app* +health promotion cloud	Standard care	Research apps	SRT	Goals and planning, feedback and monitoring, shaping knowledge, and reward and threat	24.48	TPA
Seah and Koh [41]	Adolescent girls	14.9 (+0.3 or –0.3)	13/23	*MapMyFitness* app	No intervention	Commercial apps	SDT	Goals and planning, feedback and monitoring, and social support	2.3	TPA and MVPA
Stasinaki et al [42]	Adolescents with obesity	10-18	18/13	Nutritional education and PA+PathMate2 app	Nutritional education and PA	Research apps	Theory of motivation	Goals and planning, feedback and monitoring, reward and threat, and comparison of behavior	22	CRF, WC, muscular power, muscular endurance, agility, flexibility, and balance
Tugault-Lafleur et al [43]	Children with overweight or obesity	10-17	107/107	*Aim2Be* app	No intervention	Research apps	SCT and SRT	Goals and planning, feedback and monitoring, identity, and social support	12	TPA and SB
Han et al [44]	Preschool children	3-6	66/44	YOUXUE UP app	No intervention	Research apps	Socioecological model	Goals and planning, social support, and reward and threat	8	SB, MVPA, muscle strength, muscular power, agility, flexibility, coordination, and balance
Oh et al [45]	Adolescents with obesity	13.2 (+3.6 or –3.6)	12/12	*SUKIA* app	Nintendo Switch	Research apps	NR	Feedback and monitoring and shaping knowledge	3	CRF and BMI
Staiano et al [46]	Preschoolers	4.0 (+0.8 or –0.8)	32/37	Motor skills app	Free Play app	Research apps	SCT	Goals and planning, social support, shaping knowledge, and feedback and monitoring	12	TPA, SB and MVPA
Mateo-Orcajada et al [47]	Adolescents	13.96 (+1.21 or –1.21)	240/160	*Poksammon Go* app or *Pacer* app or *Strava* app or *MapMyWalk* app	No intervention	Commercial apps	NR	8-10 change techniques per application	10	CRF, TPA, BMI, WC, muscle strength, muscular power, muscular endurance, and flexibility
Ridgers et al [38]	Inactive adolescents	13.7 (+0.4 or –0.4)	144/131	Wrist-worn Fitbit Flex and accompanying *Fitbit* app and digital behavior change resources	No intervention	Commercial apps	CT and behavioral choice theory	Goals and planning, feedback and monitoring, and self-belief	12	TPA and MVPA
Alexandrou et al [48]	Preschool-aged children	2.5-3	270/271	Standard care+ *MINISTOP 2.0* app	Standard care	Research apps	SCT	Identity, goals and planning, shaping knowledge, and feedback and monitoring	24	SB and MVPA
Mateo-Orcajada et al [49]	Adolescents	13.66 (+1.17 or –1.17)	92/46	Group 1: *Pokémon Go Playing* app continuously; group 2: *Pokémon Go Playing* app intermittently	No intervention	Commercial apps	NR	NR	10	TPA, BM, and WC

^a^BCT: behavior change technique.

^b^SRT: self-regulation theory.

^c^CRF: cardiorespiratory fitness.

^d^TPA: total physical activity.

^e^SB: sedentary behavior.

^f^MVPA: moderate to vigorous physical activity.

^g^SDT: self-determination theory.

^h^PA: physical activity.

^i^SCT: social cognitive theory.

^j^WC: waist circumference.

^k^NR: not reported.

^l^TTM: transtheoretical model.

^m^Not available.

### Risk of Bias Assessment

**Figure 2:**
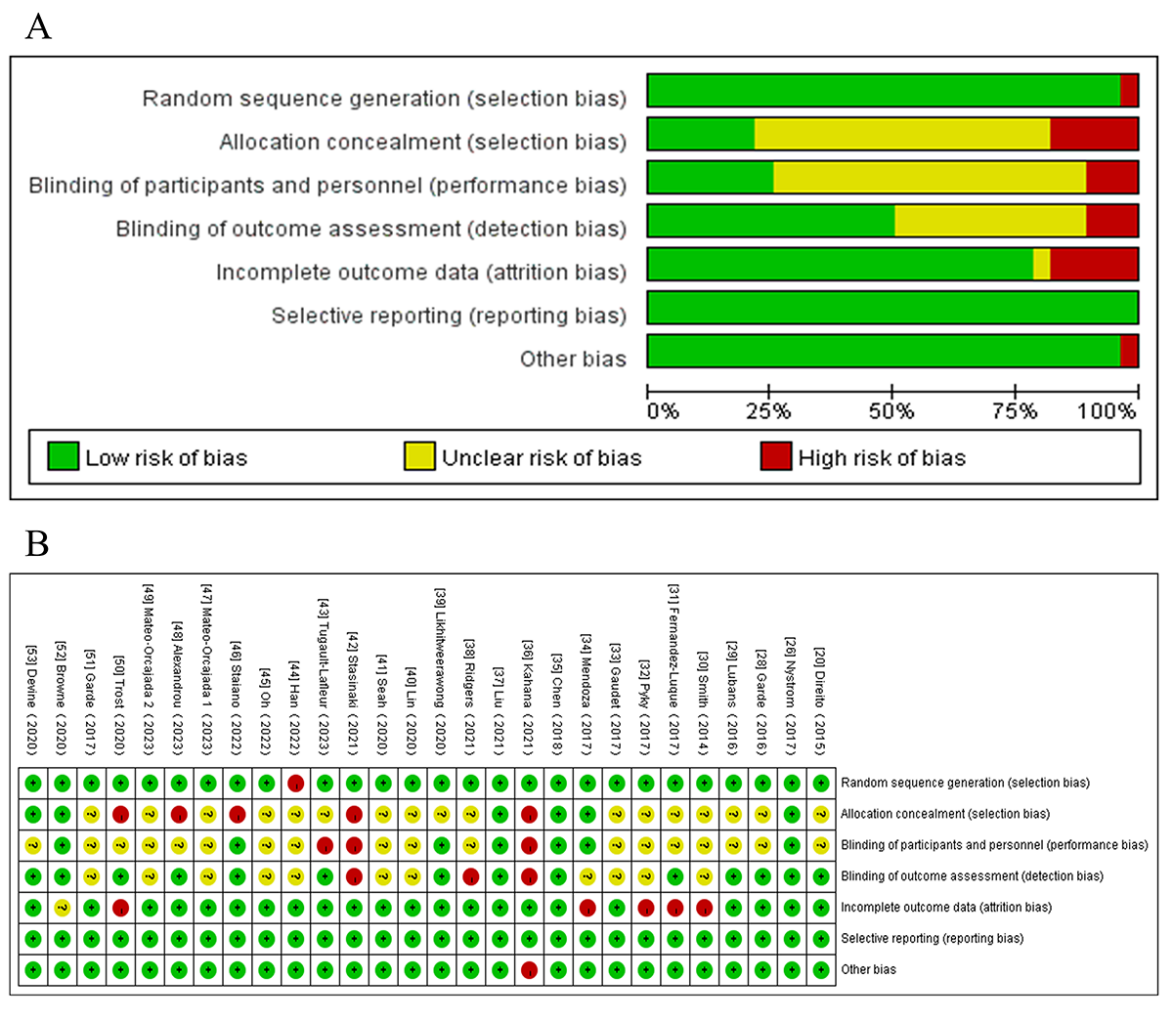
Risk bias assessment of the included studies. (A) Risk of bias graph and (B) risk of bias summary.

Overall, of the 28 studies, 11 (39%) were classified as having a low risk of bias [20,26,28,29,34,35,37,39,51-53], and a high risk of bias was identified in 17 (61%) studies [30-33,36,38,40-50]. The methods for random sequence generation were adequately reported in 28 studies, and 14 (50%) of the 28 studies described allocation concealment protocols [20,26,29,31,35,37,39,43,46,48,50-53]. Of the 28 studies, blinding of participants and personnel was unclear in 17 studies [20,28-33,38-41,43-45,47,49,51], high risk of bias was identified in 7 (25%) studies [26,34,35,37,50,52,53], and blinding of outcome assessment was unclear in 18 (64%) studies [18,28-34,38,40,41,44,45,47-51,53]. Four studies provided data regarding subjects lost to follow-up [30-32,34], while one study had unclear information on this aspect [51]. None of the 28 studies were found to have selective outcome reporting, and other aspects of bias were evaluated mainly in terms of baseline data and conflicts of interest (Figure 2 [20,26,28-53]).

### Results of the Meta-Analysis

#### Effects of mHealth App–Based Interventions on PA

**Figure 3:**
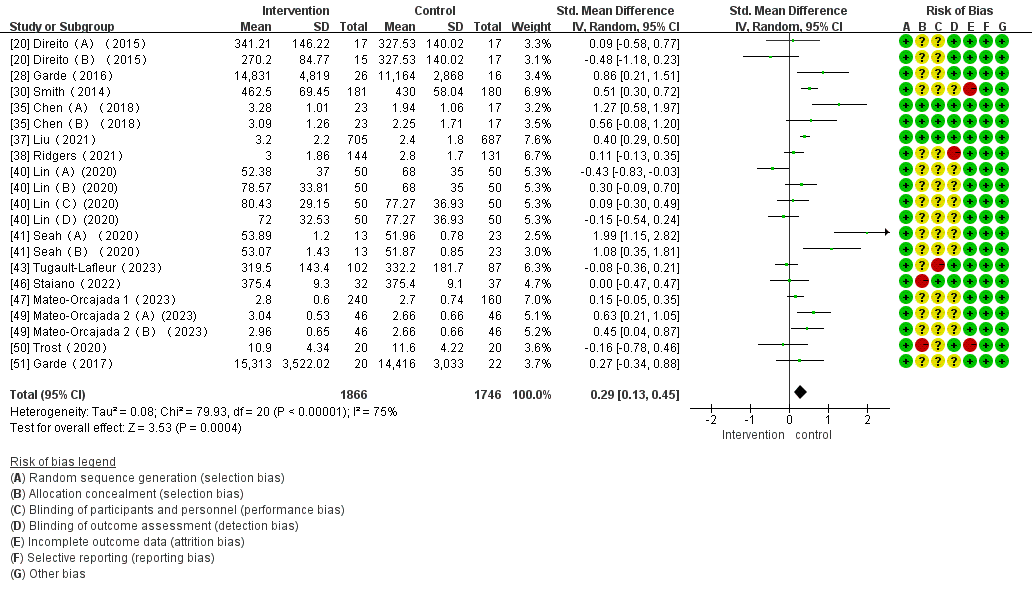
Forest plot of the effect of mobile health app–based interventions on increasing total physical activity.

Of the 28 studies, 21 (75%) were examined to assess the impact of mHealth app–based interventions on TPA. The heterogeneity test indicated a substantial level of heterogeneity among the studies (*I*^2^=75%; *P<*.001), which led to the adoption of a random effects model for the analysis. The meta-analysis results indicated that the intervention group exhibited higher TPA (SMD 0.29, 95% CI 0.13-0.45; *P*<.001; Figure 3 [20,28,30,35,37,38,40,41,43,46,47,49-51]), but the effect size was small.

**Figure 4:**
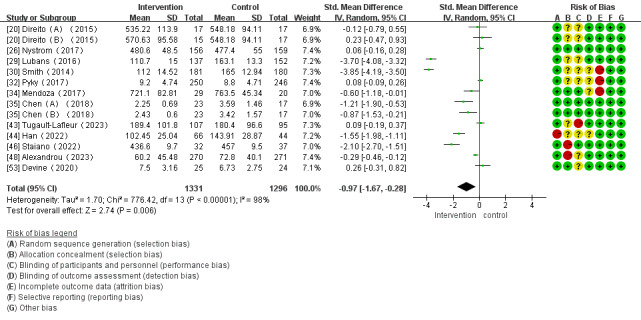
Forest plot of the effect of mobile health app–based interventions on decreasing sedentary behavior.

Of the 28 studies, 14 (50%) reported the impact of mHealth app–based interventions on SB levels. Heterogeneity tests revealed homogeneity between the studies (*I*^2^=98%; *P<*.001), which required analysis using a random effect model. Meta-analysis found that mHealth app–based interventions significantly reduced SB (SMD –0.97, 95% CI –1.67 to –0.28; *P*=.006; Figure 4 [20,26,29,30,32,34,35,43,44,46,48,53]).

**Figure 5:**
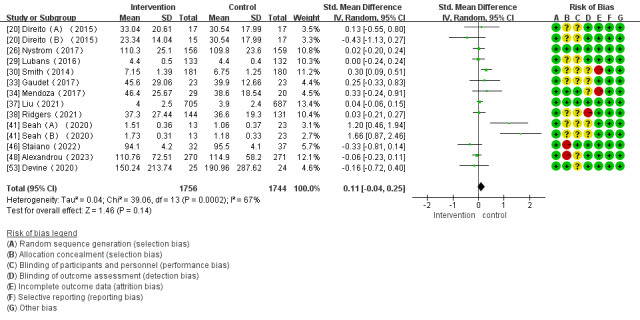
Forest plot of the effect of mobile health app–based interventions on increasing moderate to vigorous physical activity.

Of the 28 studies, 14 (50%) investigated the impact of mHealth app–based interventions on MVPA levels. The heterogeneity test indicated homogeneity among the studies (*I*^2^=67%; *P*<.001), which allowed for analysis using a random effect model. There were no significant differences between the control and intervention groups (SMD 0.11, 95% CI –0.04 to 0.25; *P*<.001; Figure 5 [20,26,29,30,33,34,37,38,41,46,48,53]).

#### Effects of mHealth App–Based Interventions on BMI and WC

**Figure 6:**
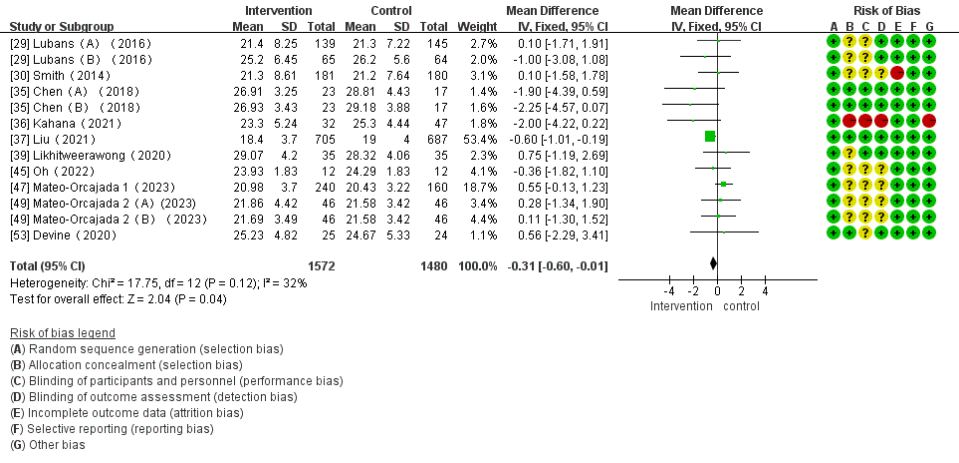
Forest plot of the effect of mobile health app–based interventions on BMI.

Of the 28 studies, 13 (46%) investigated the effects of mHealth app–based interventions on BMI. Of these 13 studies, 2 (15%) did not directly conduct changes in outcome indicators before and after interventions [31,52] and were only included in systematic reviews. The heterogeneity test demonstrated homogeneity among the studies (*I*^2^=32%; *P*=.12), which enabled analysis using a fixed-effect model. Meta-analysis found that mHealth app–based interventions significantly reduced BMI (WMD –0.31, 95% CI –0.60 to –0.01; *P*=.12; Figure 6 [29,30,35-37,39,45,47,49,53]). The 2 other studies on BMI reported a significant reduction in BMI among obese children with mHealth app–based interventions [31,52], which is consistent with the meta-analysis results.

**Figure 7:**
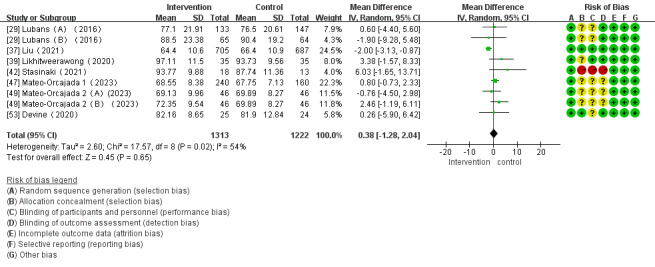
Forest plot of the effect of mobile health app–based interventions on waist circumference.

Of the 28 studies, 9 (32%) reported the effect of mHealth app–based interventions on WC. The heterogeneity tests showed homogeneity among the studies (*I*^2^=54%; *P*=.02), which allowed for analysis using a random effect model. There were no significant differences in WC between the intervention and control groups (WMD 0.38 kg/m^2^, 95% CI –1.28 to 2.04 kg/m^2^; *P*=.65; Figure 7 [29,37,39,42,47,49,53]).

#### Effects of mHealth App–Based Interventions on PF

**Figure 8:**
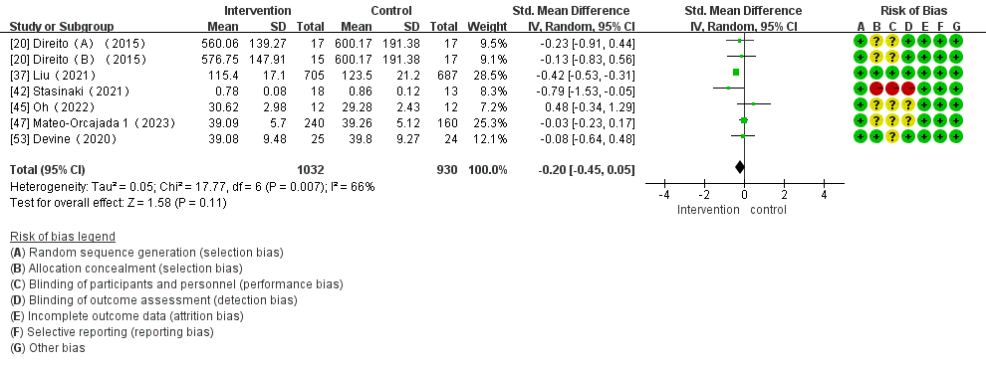
Forest plot of the effect of mobile health app–based interventions on cardiorespiratory fitness.

Of the 28 studies, 7 (25%) studies investigated the impact of mHealth app–based interventions on CRF. Heterogeneity tests showed homogeneity among the studies (*I*^2^=66%; *P*=.007) and were conducted using a random effect model. No significant differences were found between intervention and control groups in CRF (SMD –0.20 cm, 95% CI –0.45 to 0.05 cm; *P*=.11; Figure 8 [20,37,42,45,47,53]).

**Figure 9:**
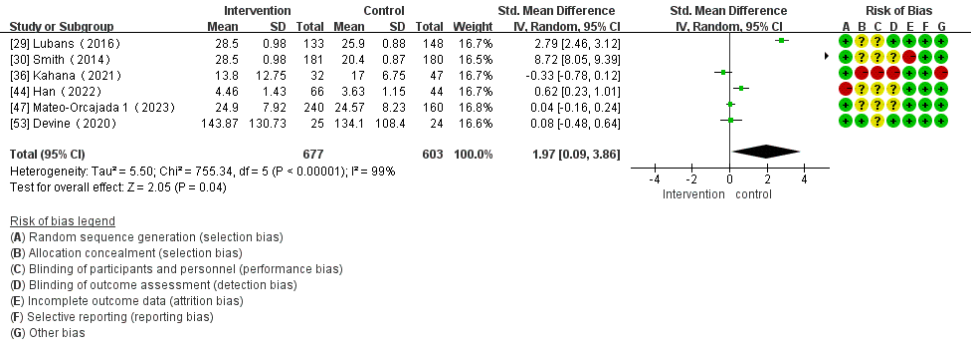
Forest plot of the effect of mobile health app–based interventions on muscular strength.

Of the 28 studies, 6 (21%) reported the impact of mHealth app–based interventions on muscle strength. Heterogeneity tests revealed homogeneity in the studies (*I*^2^=99%; *P*<.001) and were conducted using a random effect model. Meta-analysis found that mHealth app–based interventions significantly increased muscle strength (SMD 1.97, 95% CI 0.09-3.86; *P*=.04; Figure 9 [29,30,36,44,47,53]).

**Figure 10:**
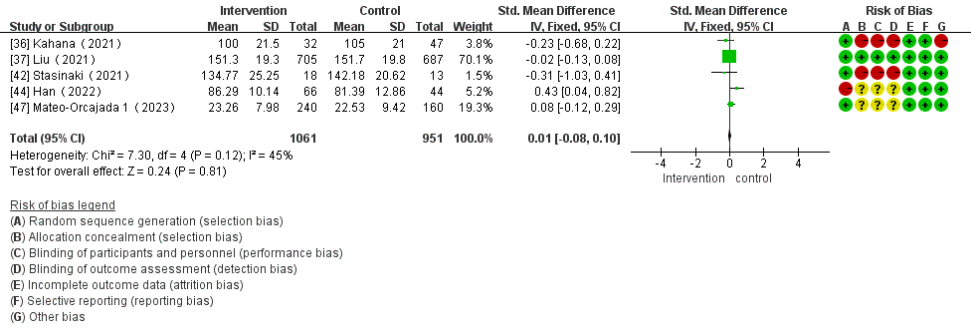
Forest plot of the effect of mobile health app–based interventions on muscular power.

Of the 28 studies, 5 (18%) investigated the impact of mHealth app–based interventions on muscular power. The heterogeneity test indicated homogeneity among the studies (*I*^2^=45%; *P*=.12), which allowed for analysis using a fixed-effect model. There were no significant differences between the control and intervention groups (SMD 0.01, 95% CI –0.08 to 0.10; *P*=.81; Figure 10 [36,37,42,44,47]).

**Figure 11:**
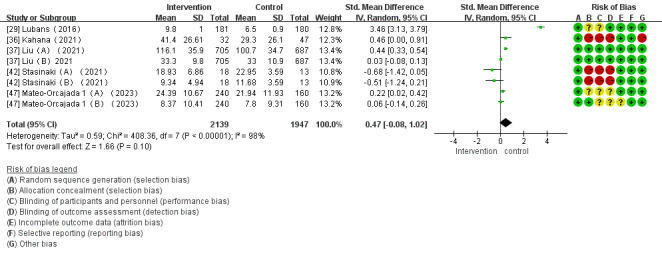
Forest plot of the effect of mobile health app–based interventions on muscular endurance.

Of the 28 studies, 5 (18%) examined the impact of mHealth app–based interventions on muscular endurance. The heterogeneity test indicated a substantial level of heterogeneity among the studies (*I*^2^=98%; *P<*.001), which led to the adoption of a random effects model for the analysis. The meta-analysis results indicated no significant difference in muscle endurance between the intervention and control groups (SMD 0.47, 95% CI –0.08 to 1.02; *P*=.10; Figure 11 [29,36,37,42,47]).

**Figure 12:**
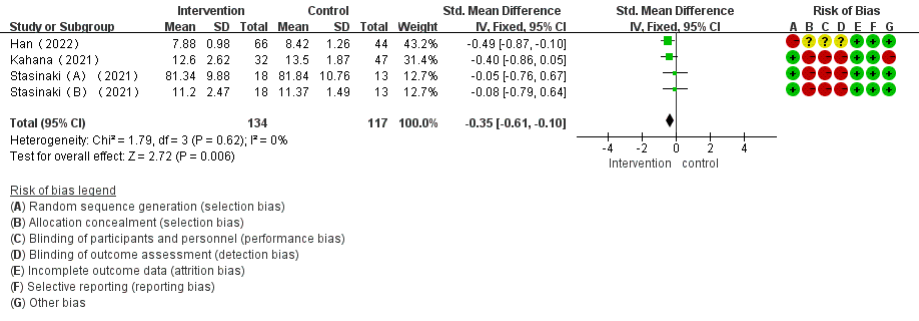
Forest plot of the effect of mobile health app–based interventions on agility.

Of the 28 studies, 3 (11%) investigated the impact of mHealth app–based interventions on agility. The heterogeneity test indicated homogeneity among the studies (*I*^2^=0%; *P*=.87), which allowed for analysis using a fixed-effect model. Meta-analysis found that mHealth app–based interventions significantly improved agility (SMD –0.35, 95% CI –0.61 to –0.10; *P*=.006; Figure 12 [36,42,44]).

**Figure 13:**
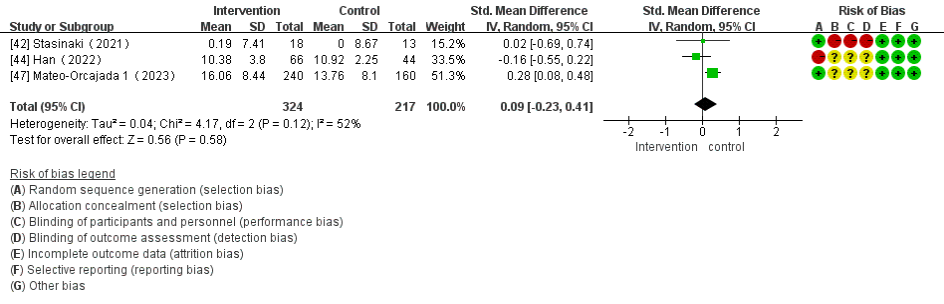
Forest plot of the effect of mobile health app–based interventions on flexibility.

Of the 28 studies, 3 (11%) reported the impact of mHealth app–based interventions on flexibility. Heterogeneity tests revealed homogeneity in the studies (*I*^2^=52%; *P*=.12) and were conducted using a random effect model. There were no significant differences in flexibility between the control and intervention groups (SMD 0.09, 95% CI –0.23 to –0.41; *P*=.58; Figure 13 [42,44,47]).

### Sensitivity Analysis

In this study, Stata 16.0 was used to conduct sensitivity analyses on TPA, SB, MVPA, and BMI for evaluating the robustness and reliability of the results. The sensitivity analysis results demonstrated that excluding any single study did not impact the effect size of the mHealth app–based intervention in outcomes such as TPA, SB, and MVPA (Multimedia Appendices 2-4), which indicates the robustness and reliability of the study results. In terms of BMI, sensitivity analyses identified 1 study as an outlier [37] (Multimedia Appendix 5). Removing this study altered the overall effect size, indicating that the study results were not sufficiently robust (SMD 0.03 cm, 95% CI –0.41 to –0.46 cm; *P*=.90) and should be interpreted with caution.

### Subgroup Analyses

#### Overview

Subgroup analyses were conducted to investigate the potential sources of heterogeneity and moderating effects, which considered factors such as age, types of apps, theoretical paradigm, number or type of BCT clusters, and intervention duration. The primary outcome indicators included TPA, BMI, SB, and MVPA. Sensitivity analysis, subgroup analysis, and assessment of publication bias were not conducted for the remaining outcome indicators due to the limited number of included studies.

#### Subgroup Analyses on the Effect of mHealth App–Based Interventions on TPA

The results of subgroup analyses investigating the impact of mHealth app–based interventions on TPA are presented in Multimedia Appendix 6. Subgroup analyses indicated no significant difference in types of intervention, theoretical paradigm, and the number of BCT clusters concerning the improvement in TPA in children and adolescents.

Age-based subgroup analysis revealed significant positive effects of interventions for children in the 7-12 years group (SMD 0.42) and adolescents in the 13-18 years group (SMD 0.29) but negligible effect for the preschool children in the 3-6 years group (SMD –0.06).

Subgroup analysis based on types of apps revealed a significantly greater effect size for the commercial apps (SMD 0.51) compared to the research apps intervention (SMD 0.13). Substantial heterogeneity existed between these 2 groups (*I*^2^=75%; *P*<.001), but the 95% CI for the effect size between the 2 groups overlapped.

Subgroup analysis based on intervention duration revealed a significant increase in TPA for the 2- to 4-week intervention (SMD 1.01) and 8- to 12-week intervention (SMD 0.23). Heterogeneity was observed between the 2 groups (*I*^2^=75%; *P*<.001), but the 95% CI for the effect size between the 2 groups overlapped.

#### Subgroup Analyses on the Effect of mHealth App–Based Interventions on SB

The results of subgroup analyses on SB are presented in Multimedia Appendix 7. Subgroup analyses showed no significant difference in intervention duration regarding the reduction of SB in children and adolescents.

Age-based subgroup analysis unveiled a notably larger effect size for interventions directed at children aged 7-12 years (SMD –3.78) compared to the group of preschool children aged 3-6 years (SMD –0.92). Heterogeneity was evident among the 2 groups (*I*^2^=99%; *P*<.001), and the 95% CI for the effect size between the 2 groups did not overlap.

Subgroup analysis based on the types of apps demonstrated a significantly stronger effect size for research app interventions (SMD –1.38) than for commercial app interventions (SMD –0.35). Heterogeneity was observed between the 2 groups (*I*^2^=98%; *P*<.001), but the 95% CI for the effect size between the 2 groups overlapped.

Subgroup analysis based on types of intervention revealed a significantly greater effect size for the concerted intervention (SMD –1.47) compared to the stand-alone apps intervention (SMD –0.45). Substantial heterogeneity existed between these 2 groups (*I*^2^=98%; *P*<.001), but the 95% CI for the effect size between the 2 groups overlapped.

Subgroup analyses based on the theoretical paradigm showed a greater effect size for interventions based on the SCT (SMD –0.64) than for interventions based on combination of SCT and other theories (SMD –2.18) and interventions solely based on SRT (SMD 0.05). Substantial heterogeneity existed between these 2 groups (*I*^2^=98%; *P*<.001), but the 95% CI for the effect size between the 2 groups overlapped.

Subgroup analysis based on the number of BCT clusters demonstrated a significant reduction in SB for interventions based on 7-10 BCT clusters (SMD –1.03) and interventions based on 4 BCT clusters (SMD –1.36), and the 95% CI for the effect size between the 2 groups overlapped.

Subgroup analysis based on intervention duration revealed a significant reduction in SB for the 20- to 48-week intervention (SMD –1.42) but had a negligible effect at the 8- to 12-week intervention (SMD –0.63).

#### Subgroup Analyses on the Effect of mHealth App–Based Interventions on MVPA

The results of subgroup analyses on MVPA are shown in Multimedia Appendix 8. Subgroup analyses revealed no significant difference in type of apps, types of intervention, and the number of BCT clusters in improving MVPA in children and adolescents, which were not moderators of the effect of mHealth app interventions.

Age-based subgroup analyses unveiled a notably larger effect size for interventions directed at adolescents aged 13-18 years (SMD 0.42) compared to the group of preschool children aged 3-6 years (SMD –0.05) and children aged 7-12 years (SMD 0.11). Heterogeneity was evident among the 2 groups (*I*^2^=68%; *P*<.001), but the 95% CI for the effect size between the 2 groups overlapped.

Subgroup analyses based on theoretical paradigm demonstrated a significant increase in MVPA for SDT-based intervention (SMD 1.03), while no significant effect was observed for SCT (SMD –0.06), combination of SCT and other theories (SMD 0.12), and SRT (SMD –0.14).

Subgroup analysis based on intervention duration revealed a significant increase in MVPA for the 2- to 4-week intervention (SMD 1.42) but negligible effect for the 8- to 12-week intervention (SMD –0.01) and 20- to 48-week intervention (SMD 0.05).

#### Subgroup Analyses on the Effect of mHealth App–Based Interventions on BMI

The results of the subgroup analyses based on BMI are shown in Multimedia Appendix 9. Subgroup analyses revealed no significant difference in type of apps, theoretical paradigm, and the number of BCT clusters in decreasing BMI in children and adolescents.

Age-based subgroup analysis revealed a significant decrease in BMI for interventions targeting children aged 7-12 years (WMD –0.59), but no significant difference was observed for adolescents aged 13-18 years (WMD 0.03).

Subgroup analysis based on types of intervention revealed a significantly greater effect size for the concerted intervention (WMD –0.59) compared to the stand-alone apps intervention (WMD 0.34), but the 95% CI for the effect size between the 2 groups overlapped.

Subgroup analysis based on intervention duration revealed a significant reduction in BMI for the 20- to 48-week intervention (WMD –0.57) and the 8- to 12-week intervention (WMD 0.33), and the former showed a clearly superior effect.

### Meta-Regression

We conducted meta-regressions for TPA, BMI, SB, and MVPA, focusing on statistically significant moderators identified in the subgroup analyses. The results of the meta-regression for TPA (*P*=.03, 95% CI –1.127 to –0.082) and MVPA (*P*=.045, 95% CI –2.052 to –0.033) revealed that intervention duration had a potential moderating effect on high heterogeneity; no statistical differences were found for other variables acting as moderators. In the case of BMI, meta-regression results indicated that age (*P*=.04, 95% CI 0.041-1.473) and types of intervention (*P*=.02, 95% CI –1.648 to –0.205) could be potential moderators for high heterogeneity, whereas other factors did not significantly contribute to explaining high heterogeneity.

### Reporting Biases

Funnel plots were used to assess publication bias in the effects of mHealth app–based interventions on TPA, SB, BMI, and MVPA in children and adolescents. The funnel plots exhibited mostly symmetrical patterns in the 4 studies (Multimedia Appendices 10-13). In addition, Egger test was performed for TPA (*t*_20_=0.01; *P*=.99), SB (*t*_13_=–0.135; *P*=.20), MVPA (*t*_13_=1.22; *P*=.25), and BMI (*t*_12_=–0.07; *P*=.95), and the results suggested no significant publication bias (Multimedia Appendices 14-17).

## Discussion

### Principal Findings

We conducted a systematic review and meta-analysis to assess the effectiveness of mHealth app–based interventions in promoting PA and enhancing PF in children and adolescents. This study also examined the potential moderators that may influence the efficacy of these interventions. The findings of this study suggest that mHealth app–based interventions may yield positive effects on TPA, SB, BMI, agility, and muscle strength in children and adolescents. However, no significant effects were observed for MVPA, WC, CRF, muscular power and endurance, and flexibility. Age, theoretical paradigm, BCT clusters, types of intervention, types of apps, and intervention duration were identified as significant moderating factors associated with the increased effectiveness of mHealth app interventions on PA and PF, but the impact on effect size is not entirely consistent.

### Overall Effect

#### PA Levels

The findings of this study indicated that mHealth app–based interventions increased TPA and reduced SB among children and adolescents but had no significant effect on MVPA. Our research findings represent a valuable expansion of recently published systematic reviews; however, they do not entirely align with the results of previous studies. A prior systematic review reported that mHealth-based interventions increased TPA levels and addressed physical inactivity in children and adolescents but did not lead to reduced SB and improved MVPA [12,13]. The inconsistency in the findings may be attributed to the cointervention effect of technologies such as SMS text messaging, wearable devices, web-based interventions, and smartphone apps [13]. Among these, smartphone app–based interventions might be the most effective strategy. The use of apps may contribute to increased SB time. Nevertheless, results of this study indicate that mHealth app–based interventions can effectively reduce SB in children and adolescents. Distinguishing whether the effect is from stand-alone app interventions or other strategies within concerted interventions is challenging. Subgroup analyses in this study demonstrated the superiority of concerted interventions over stand-alone app interventions. In conclusion, mHealth app–based interventions serve as a valuable adjunct in reducing SB in children and adolescents. Further concerted interventions, such as combining educational policies with mHealth apps interventions, are recommended.

The effectiveness of mHealth app–based interventions was influenced by age and intervention format. Notably, these interventions were more effective in improving PA levels among adolescents compared to children, and greater effects were observed when using mHealth apps than when using only SMS text messaging interventions [35,50]. These findings support the conclusions of this study, but some researchers hold differing views. The study by Trost and Brooks [50] indicated that an 8-week intervention with Moovosity apps (Kinetica Group Pty Ltd) improved proficiency in fundamental movement skills (FMS) but did not increase PA levels. The phenomenon may be associated with the types of design and goal setting of the apps. The apps in the study were primarily used to increase the FMS of children, which may have resulted in the activity of FMS replacing the original PA. As a result, the TPA of children was unchanged. Another study [35] identified that targeted app interventions would be effective in reducing SB in adolescents, which must be based on certain theoretical paradigms and BCT clusters. Gamification-based app interventions are also more favorable for increasing TPA and reducing SB levels in children and adolescents and must be combined with the theoretical paradigm, intervention duration, and features of apps [55]. In conclusion, the reasons for inconsistent intervention results may be related to population characteristics, types of apps, theoretical paradigm, BCT clusters, and intervention duration. Moreover, further research is needed in the future.

#### PF Levels

Another significant finding of the meta-analysis was that mHealth app–based interventions decreased BMI and improved muscle strength and agility among children and adolescents. However, no significant effects were observed for WC, CRF, muscular power and endurance, and flexibility. These findings are not entirely aligned with the results of previous studies. For example, a previous study reported an increase in PA levels among adolescents and significant reductions in sugary drink consumption and BMI following a 3-month intervention using Fitbit Flex apps (Fitbit, Inc) [35]. Meanwhile, a different study [29] indicated that an 18-month app intervention did not lead to significant reductions in BMI and WC, but it improved exercise capacity and reduced screen time. Several potential factors may contribute to the inconsistent results. First, the choice of outcome measure could be a contributing factor. BMI and WC may indicate distinct aspects of obesity; although BMI primarily assesses body size and shape, WC is a measure of abdominal obesity, leading to a weak correlation between the two [56,57]. Moreover, BMI tends to underestimate overweight prevalence when abdominal obesity is considered [57]. Hence, although mHealth app–based interventions show minor effects on BMI in children and adolescents, they might not induce significant changes in WC. Nonetheless, such interventions can still yield favorable outcomes. Another possible reason for the inconsistency in interventions could be related to the age of the population under study. Some studies have shown that mHealth app–based interventions are effective in reducing body weight and BMI in individuals aged ≥45 years, but they are less effective in children and adolescents [58]. In addition, our meta-regression analysis revealed that age significantly influences the effectiveness of app**-**based interventions. This result could be attributed to the fact that children and adolescents are in a period of rapid growth and development, and the effects of the interventions may be masked by the significant changes in height and weight during this stage.

In terms of muscular strength, power, and endurance, previous studies have shown that a 6-month intervention using mHealth apps and trackers resulted in increased muscle strength among children and adolescents but did not have a significant effect on CRF [53]; Stasiak et al [42] discovered that a daily conversational agent intervention, using a mobile app for adolescents with obesity, enhanced subjects’ muscular fitness, with no discernible difference between the intervention and control groups. Conversely, other studies have indicated that interventions using AIMFIT apps can positively impact maximal oxygen uptake [20]; Mateo-Orcajada et al [47] found that a 10-week after-school intervention for adolescents using mobile step-tracking apps significantly enhanced subjects’ upper limb strength, hamstring and lower back flexibility, explosive power of the lower limbs, as well as abdominal muscular strength and endurance. Various factors can influence the effectiveness of interventions, including subject characteristics, intervention dosage, engagement levels, and willingness to use apps. Some of the studies included in this work involved participants who were patients with cancer [53] or children with overweight and obesity [42], and this factor may have influenced the outcomes of the intervention. Furthermore, low user willingness to engage with apps may reduce PA engagement, which consequently impacts the effectiveness of the intervention. Finally, insufficient dosage of mHealth app–based interventions has been associated with a small effect size on TPA in children and adolescents and has shown no increase in MVPA levels. This insufficiency in dosage may explain the lack of intervention effects observed in children.

#### Moderating Variables on the Effects of mHealth App–Based Interventions for PA and PF

#### Age

The study results revealed that age moderates the effects of mHealth app interventions on TPA, MVPA, SB, and BMI. Subgroup analysis indicated that mHealth app–based interventions significantly increased TPA and MVPA and reduced SB in children and adolescents aged 7-18 years; increased TPA and MVPA in adolescents aged 7-18 years; and only reduced SB in children aged 3-6 years. It is noteworthy that previous studies have indicated that mHealth app–based interventions did not effectively decrease SB in children and adolescents. This observation may stem from the fact that the use of apps may result in increased SB time, consequently reallocating the time resources of children and adolescents [12]. However, the findings of this study do not support this conclusion. A separate study reported that the intervention using Fit Survivor apps on SB in adolescents was ineffective, which is possibly due to the characteristics of the population; notably, most participants in these studies had cancer, which may require a longer period of SB to observe significant changes [53]. The findings regarding BMI indicate that parent-focused mHealth app–based interventions have not been successful in reducing BMI among children and adolescents [29], somewhat not entirely consistent with the findings of this study. Our meta-regression analysis revealed that age significantly influences the effectiveness of app-based interventions.

This phenomenon may be closely associated with willingness of users and their behavioral intention to use apps. Individual differences, such as age and gender, play a crucial role in moderating willingness and intention of users. In general, behavioral habits and expected performance serve as predictors of willingness and intention of users [59], and age can increase the frequency of use by moderating behavioral habit and performance [60], which in turn have an influence on SB. In addition, most of the app intervention is parenting-focused intervention among preschoolers [61]. The successful implementation of interventions relies on parental involvement, thus parental attitudes significantly influence the participation of preschoolers aged 3-6 years, potentially impacting the efficacy of mHealth app interventions [13]. In the case of children and adolescents aged 7-18 years, increasing age correlates with improved expected performance [62]. In adolescents, this performance expectation is closely linked to user intention, bolstering their willingness to engage with apps. However, older adolescents, who prioritize academic achievement, may exhibit reduced effectiveness in app interventions [63]. In conclusion, the effects of mHealth app–based interventions may vary across different age groups. However, only 4 studies included subjects aged 3-6 years, potentially affecting the generalizability of the findings. Therefore, further validation through a larger number of high-quality studies is warranted to strengthen the conclusions.

#### Types of Intervention

Concerning types of interventions, there is a lack of systematic reviews analyzing the impacts of stand-alone apps and concerted interventions on PA and PF, including their distinctions. Subgroup analysis revealed that concerted interventions significantly increased TPA and reduced SB and BMI in children and adolescents, whereas stand-alone app interventions had only a modest positive effect on TPA with a small effect size. The findings diverge somewhat from prior research. Some studies indicate that interventions using COOL Passport apps or a combination of COOL Passport apps and game-based interactive platforms do not enhance PA in youth with congenital heart disease [40]. Conversely, other studies suggest that mobile apps can increase TPA while decreasing SB and BMI in youth [46,47]. This phenomenon may be associated with behavior change interventions. Effective behavior change interventions can enhance health status and reduce health care spending, with theory-based interventions often yielding superior results [64]. Hence, interventions based on the high-scoring theoretical paradigm, or in combination with other specific high-scoring strategies, are more likely to yield positive outcomes [65]. In summary, interventions integrating apps with high-scoring strategies are probably more effective than stand-alone apps, although the optimal number and type of strategies remain undetermined.

#### Theoretical Paradigm

Effective behavior change interventions can improve health status and reduce health care spending, and theory-based interventions generally yielded superior results [64]. Currently, mHealth app interventions use multiple types of BCT that have been shown to be effective in influencing intervention outcomes [21]. Of the 28 studies, 8 (29%) did not use theoretical frameworks in their mHealth app interventions [31,36,37,45,47,49,50,52], while the remaining 20 (71%) studies were grounded in theoretical paradigms such as SCT, SDT, and SRT.

The most commonly used theoretical paradigms in the research are SCT and SDT, and interventions based on these frameworks have demonstrated positive effects on TPA, MVPA, and SB in children and adolescents. Subgroup analysis revealed that interventions based on SDT significantly increased TPA and MVPA in children and adolescents, while interventions based on SCT significantly reduced SB in this population. Previous studies have consistently shown that interventions based on SCT effectively improve PA among adolescents, which aligns with the findings of this study [54]. SCT is commonly used to elucidate the mechanisms underlying the improvement in PA through behavior change interventions. SCT posits that PA is influenced by personal, social, and environmental factors. Personal factors, including self-efficacy, self-management, and expected performance, play a crucial role in enhancing PA and reducing SB. mHealth apps can effectively promote the increase in PA and the reduction in SB by modifying these factors [66].

SDT is frequently used to account for variations in individual behavior resulting from differences in intrinsic and extrinsic motivation. Intrinsic motivation, which is a crucial predictor of PA levels, is lacking in approximately 40% of Europeans; this situation leads to failure to meet the recommended PA levels [67]. In contrast to extrinsic motivation, intrinsic motivation drives the initiation and sustained engagement in individual behaviors. Thus, interventions grounded in SDT can effectively enhance leisure-time PA and PA in physical education classes and reduce SB among adolescents by fostering intrinsic motivation [68]. Furthermore, interventions using mHealth apps based on the health belief model and theory of planned behavior may yield positive effects on increasing PA in children and adolescents. However, which theory yields optimal intervention effects requires further investigation. In conclusion, interventions based on SCT or SDT within mHealth apps may likely yield more effective results. However, due to the limited number of included studies, further validation through a larger volume of high-quality research is necessary to bolster these conclusions.

#### BCT Clusters

BCT clusters represent specific implementation strategies in behavior change interventions. Previous studies have demonstrated that interventions incorporating a specific number of BCT clusters are more effective in promoting the PA of users. However, further investigation is needed to determine the optimal combination of the number and type of BCT clusters to achieve optimal intervention effects [69].

The BCT clusters that emerged frequently in this study were feedback and monitoring (n=22), goals and planning (n=19), shaping knowledge (n=12), and social support (n=11). A previous study [70] focusing on app interventions targeting PA and SB in adults also identified feedback and monitoring as well as goals and planning as the most commonly used BCT clusters. Another study [16] also highlighted the positive effects of mHealth app–based interventions on PA and SB, particularly when specific BCT clusters such as goals and planning, feedback and monitoring, and social support were implemented. Consequently, specific BCT clusters, including goals and planning, feedback, and monitoring, play a crucial role in influencing the effectiveness of mHealth app–based interventions, ensuring increased TPA, decreased SB, and reduced BMI by enhancing the willingness and engagement of users [71].

Limited research exists on the impact of varying numbers of BCT clusters implemented on PA and PF in children and adolescents. In this study, we discovered that mHealth app–based interventions incorporating 4 and 7-10 BCT clusters resulted in a significant reduction in SB among child adolescents, and the latter showed a greater effect size than the former. Conroy et al [72] reported a mean of 4.2 for the use of BCT in app. The number of studies included in the analysis varies, and most studies used 4 BCT clusters, which is slightly lower than the number of BCT used in previous studies. This discrepancy may be attributed to the characteristics of the subjects [73]. Moreover, more BCT clusters in mHealth apps do not necessarily lead to better outcomes. Excessive use of BCT can decrease the willingness of users and their frequency of app use [74]. Thus, further research is needed to determine the optimal number of BCT clusters.

This phenomenon appears to be strongly associated with the willingness of users and their engagement in using apps. Feedback and monitoring, which involves design patterns enabling users to track their performance or status, play a crucial role in enhancing the trust, motivation, and engagement with the app of users. Consequently, this situation leads to the effective promotion of PA levels and reduction in BMI [75]. Similarly, goals and planning, which involve planned behaviors and the conversion of the intentions of users into actionable steps through self-regulation and self-efficacy, have been shown to improve PA and reduce BMI [76].

#### Types of Apps and Intervention Duration

The available evidence suggests that commercial apps did not yield improvements in PA or reductions in SB among children and adolescents [20,31-33,35]. However, Jasmine et al [77] found that integrating commercial apps with web-based social networking platforms was effective in improving PA. However, a certain use frequency was needed, and a significant correlation between frequency of use and PA was observed. Our study discovered that research apps significantly reduced SB, while commercial apps increased TPA in children and adolescents, but the effect size was small. However, the effect size was small for commercial apps, which contrasts with our expectations. This discrepancy may be attributed to the design and characteristics of the apps. Commercial app developers often prioritize user interface simplification and the inclusion of complex features to enhance app engagement. However, they may overlook incorporating theoretical frameworks and BCT clusters that are closely associated with intervention effectiveness [78].

An intriguing finding emerged regarding the duration of the interventions. The 2- to 4-week and 8- to 12-week interventions significantly increased TPA, but the former demonstrated superior effectiveness to the latter; 8- to 12-week interventions significantly increased MVPA. Furthermore, the 20- to 48-week intervention significantly reduced SB and BMI in children and adolescents. This finding aligns with the results of previous studies that short-term mHealth app interventions effectively increased the PA of children, while longer interventions yielded diminished outcomes [40,51]. The results of this study support the notion that intervention effects are closely linked to the willingness of participants to engage. The reason is that shortly after engaging in an mHealth app–based intervention, children and adolescents will experience a sensitive attraction period [79]; prolonged interventions may lead to diminished interest and compliance, thereby weakening the impact. Another plausible explanation is that behavior change interventions rooted in theoretical frameworks yield better outcomes, but they require sufficient time for users to develop habitual behaviors conducive to lasting positive changes, and interventions targeting SB and BMI take even longer. In addition, our meta-regression analysis revealed that intervention duration significantly influences the effectiveness of app**-**based interventions.

### Limitations

This study has several limitations. The search was restricted to English literature, which may introduce language bias and affect the reliability of the findings. Only a limited number of published articles were included, which potentially omit unpublished manuscripts and other sources (eg, conference papers and dissertations) that could contribute to publication bias. The outcome measures used in this study had inconsistent units, and the interpretation of the results using SMD as effect indicators requires caution. PF encompasses multiple dimensions, and different studies focused on various subdimensions.

Most studies (21/28, 75%) encompassed multiple metrics, and some studies (11/28, 39%) were not primarily designed to enhance PA and PF. These factors could have biased the results, potentially limiting the effect sizes and hindering a full reflection of the true effects in this study. Despite our extensive meta-regression and subgroup analyses, only age and intervention period demonstrated significant associations. Heterogeneity for some outcome metrics remained high, and the 95% CIs for the effect size overlapped between groups. Consequently, caution is advised when interpreting partial conclusions from subgroups. Our findings indicate heterogeneity in some studies, and the source of this heterogeneity remains unclear. The effectiveness of mHealth app–based interventions may vary among children and adolescents based on demographics (eg, ethnic backgrounds, regions, genders, and BMI), economic levels, intervention models (parent centered vs child centered), and the degree of app individualization. These factors could contribute to the observed heterogeneity. However, due to limitations stemming from the number and characteristics of included studies, these factors were not analyzed in this study. In conclusion, the findings of this systematic review and meta-analysis should be interpreted cautiously.

### Conclusions

The findings from a systematic review and meta-analysis indicate that mHealth app–based interventions hold significant potential as a therapeutic strategy for increasing TPA levels, reducing BMI and SB, and improving agility and muscle strength among children and adolescents. However, these interventions insignificantly affected MVPA, WC, CRF, muscular power and endurance, and flexibility, which are crucial for promoting PA and enhancing PF. Age, app types, types of intervention, theoretical paradigms, BCT clusters, and intervention duration emerged as important moderating variables that influence the effectiveness of mHealth app–based interventions. These moderating effects should be considered during the design, preparation, and promotion of interventions.

Considering the potential benefits of using mHealth apps, future research should explore the combination of different types and quantities of mHealth app–based interventions to determine the optimal approach for increasing PA levels and improving PF in children and adolescents. Furthermore, future studies could investigate the impact of additional influences on the intervention effectiveness of mHealth app interventions, such as demographics characteristics, economic levels, and intervention models.

## Appendix

Multimedia Appendix 1 Literature search strategy.

Multimedia Appendix 2 Sensitivity analyses results on total physical activity.

Multimedia Appendix 3 Sensitivity analyses results on sedentary behavior.

Multimedia Appendix 4 Sensitivity analyses results on moderate to vigorous physical activity.

Multimedia Appendix 5 Sensitivity analyses results on BMI.

Multimedia Appendix 6 Summary of subgroup analysis results of mobile health app–based interventions on total physical activity.

Multimedia Appendix 7 Summary of subgroup analysis results of mobile health app–based interventions on sedentary behavior.

Multimedia Appendix 8 Summary of subgroup analysis results of mobile health app–based interventions on moderate to vigorous physical activity.

Multimedia Appendix 9 Summary of subgroup analysis results of mobile health app–based interventions on BMI.

Multimedia Appendix 10 Funnel plot of total physical activity.

Multimedia Appendix 11 Funnel plot of sedentary behavior.

Multimedia Appendix 12 Funnel plot of BMI.

Multimedia Appendix 13 Funnel plot of moderate to vigorous physical activity.

Multimedia Appendix 14 Egger test results on total physical activity.

Multimedia Appendix 15 Egger test results sedentary behavior.

Multimedia Appendix 16 Egger test results moderate to vigorous physical activity.

Multimedia Appendix 17 Egger test results BMI.

Multimedia Appendix 18 PRISMA 2020 Checklist.

## Abbreviations

BCT: behavior change technique
CRF: cardiorespiratory fitness
FMS: fundamental movement skills
mHealth: mobile health
MVPA: moderate to vigorous physical activity
PA: physical activity
PF: physical fitness
PRISMA: Preferred Reporting Items for Systematic Reviews and Meta-Analyses
RCT: randomized controlled trial
SB: sedentary behavior
SCT: social cognitive theory
SDT: self-determination theory
SMD: standardized mean difference
SRT: self-regulation theory
TPA: total physical activity
WC: waist circumference
WMD: weighted mean difference
