# Supplementary material for: Effectiveness of mHealth App–Based Interventions for Increasing Physical Activity and Improving Physical Fitness in Children and Adolescents: Systematic Review and Meta-Analysis
Source: JMIR Mhealth Uhealth. 2024 Apr 30;12:e51478. doi: 10.2196/51478 (PMC11094610; doi:10.2196/51478)
Supplement: Multimedia Appendix 1 [file mhealth_v12i1e51478_app1.pdf]

Multimedia Appendix 1: Literature search strategy

Database searched:Web of Science Core Collection

The following search terms were used:

Child OR Preschool OR Adolescent OR Children OR “Preschool Child” OR “Preschool Children” OR Adolescents OR Adolescence OR Teen* OR Teenager* OR Youth* OR “Female Adolescent*” OR “Male Adolescent*" (Title) and “Mobile health application*” OR “m-health app*” OR “Portable Software Application*” OR “Portable Software App*” OR “Mobile Application*” OR App* OR “Mobile App*” OR “Smartphone App*” OR “Portable Electronic App*” OR “Portable Electronic Application*” (Topic) and “Physical Activity” OR PA OR “Physical Activities” OR “moderate to vigorous physical activity” OR “moderate-to-vigorous physical activity” OR “leisure time activity” OR “leisure-time activity” OR walking OR steps OR running OR sitting OR “sedentary behavior” OR “sedentary behaviour” OR SB OR inactive OR inactivity OR “active lifestyle” OR “sedentary lifestyle” OR “Physical Fitness” OR fitness OR “Performance-Related Physical Fitness” OR “Health-Related Physical Fitness” OR “physiologic fitness” OR strength OR power OR balance OR endurance OR flexibility OR “body composition” OR “body mass index” OR BMI OR “maximal oxygen uptake” OR “maximal oxygen consumption” OR “aerobic capacity” OR VO2 max (Topic) and “Randomized Controlled Trial*” OR RCT* OR “Random control test” OR “Randomized comparison study” OR “controlled clinical trial” OR Randomized OR Randomly OR groups OR Trial OR placebo (Topic)
