# Supplementary figures and images for: Effectiveness of mHealth App–Based Interventions for Increasing Physical Activity and Improving Physical Fitness in Children and Adolescents: Systematic Review and Meta-Analysis
Source: JMIR Mhealth Uhealth. 2024 Apr 30;12:e51478. doi: 10.2196/51478 (PMC11094610; doi:10.2196/51478)

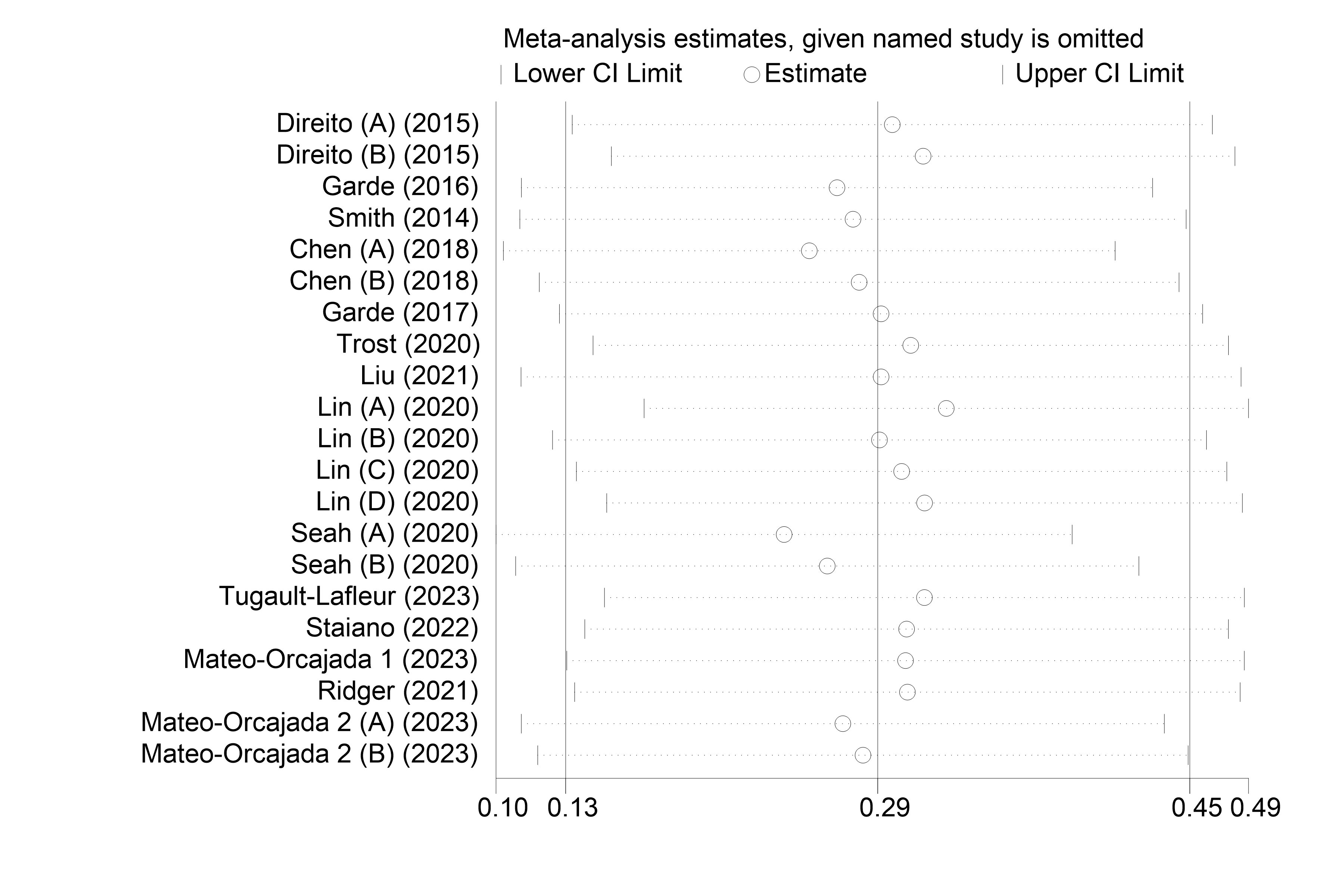

Supplement: Multimedia Appendix 2 [file mhealth_v12i1e51478_app2.png]

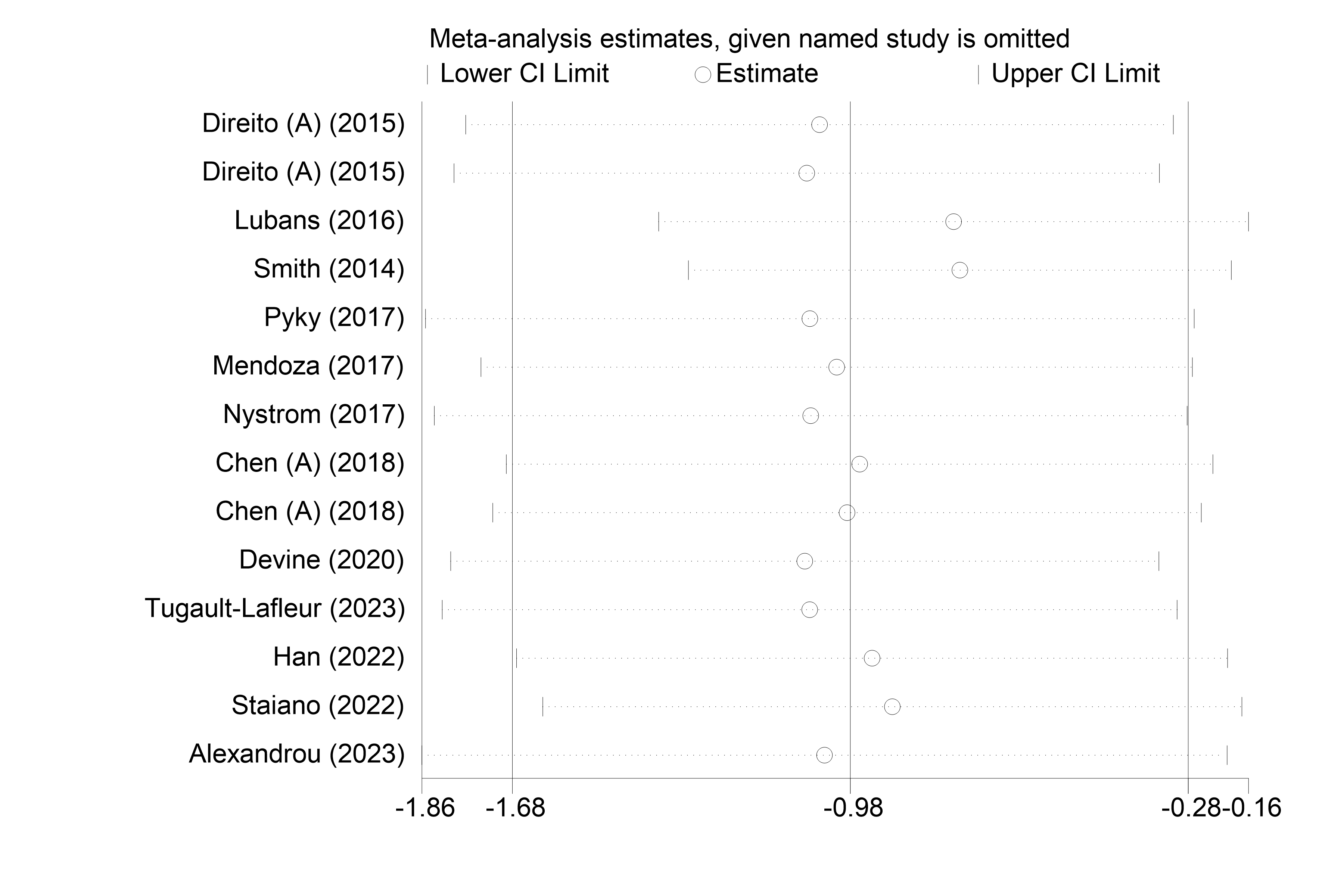

Supplement: Multimedia Appendix 3 [file mhealth_v12i1e51478_app3.png]

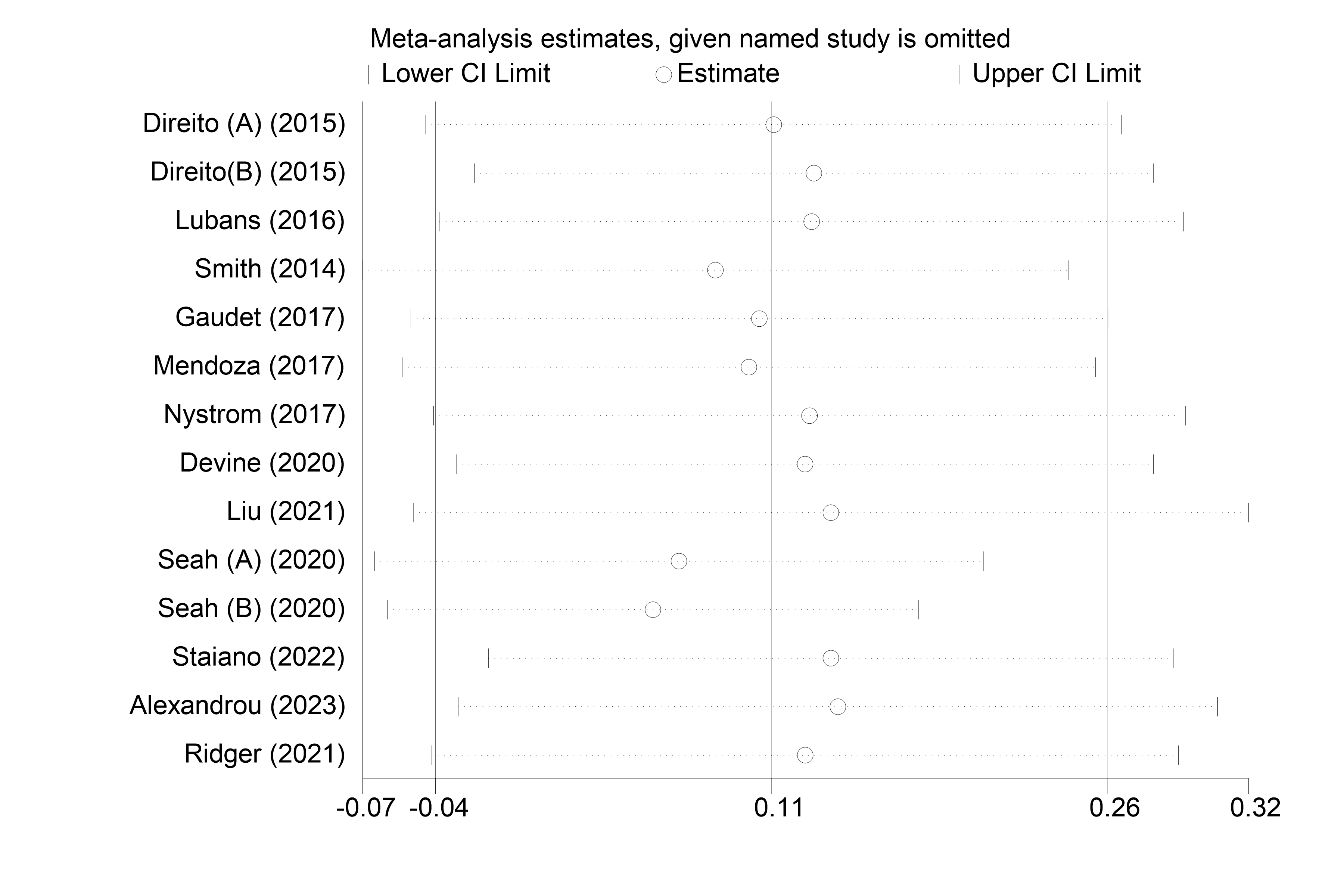

Supplement: Multimedia Appendix 4 [file mhealth_v12i1e51478_app4.png]

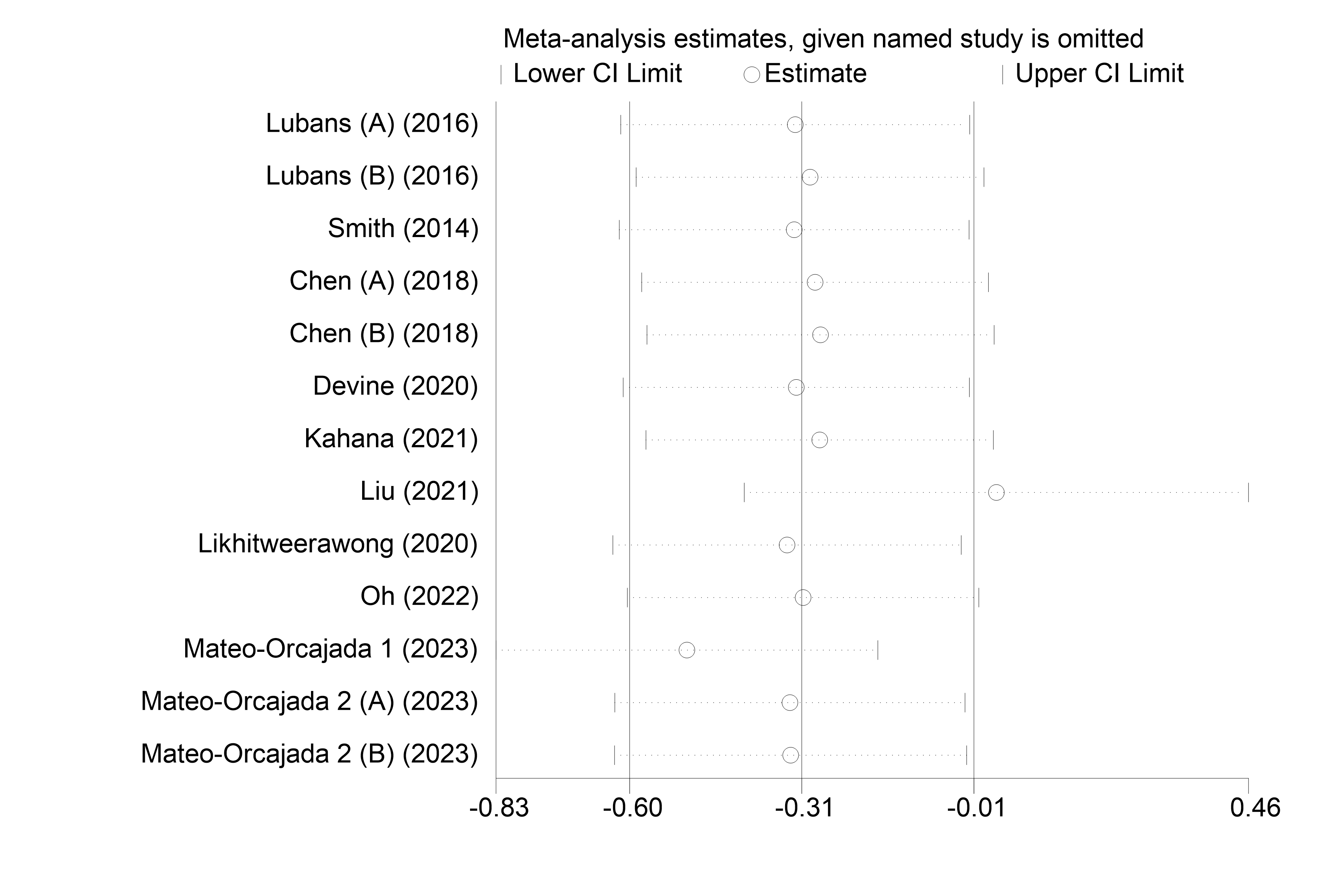

Supplement: Multimedia Appendix 5 [file mhealth_v12i1e51478_app5.png]

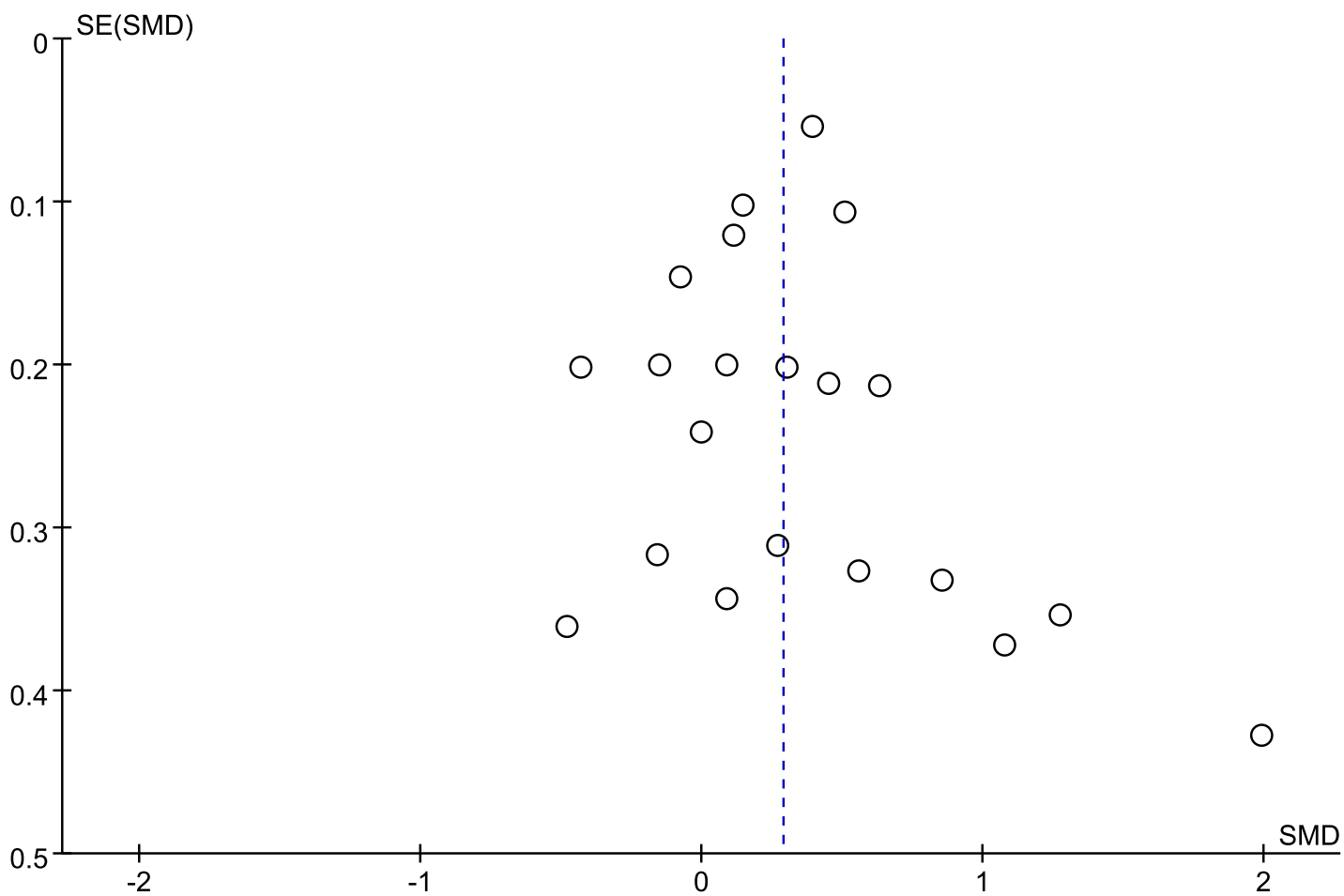

Supplement: Multimedia Appendix 10 [file mhealth_v12i1e51478_app10.pdf]

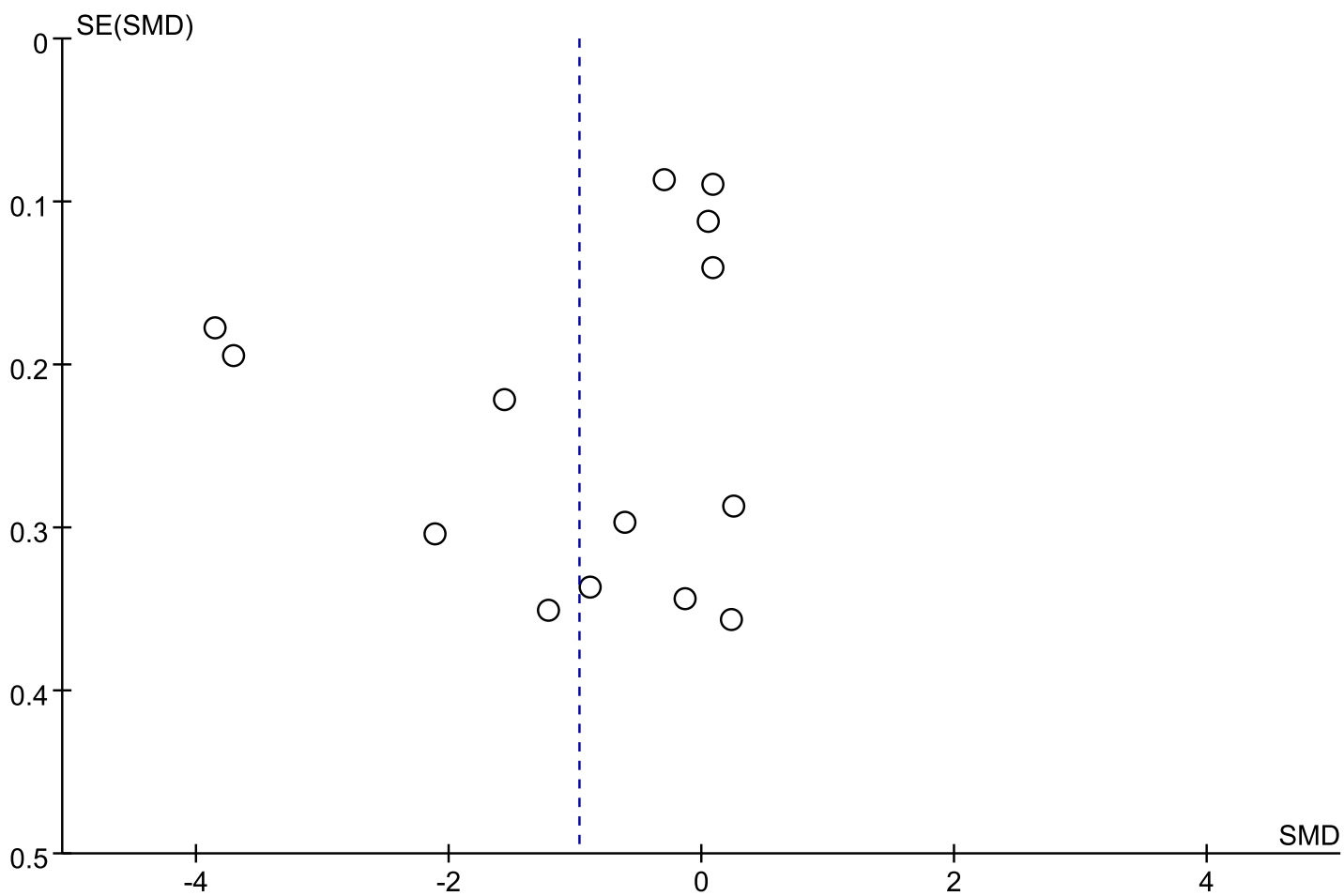

Supplement: Multimedia Appendix 11 [file mhealth_v12i1e51478_app11.pdf]

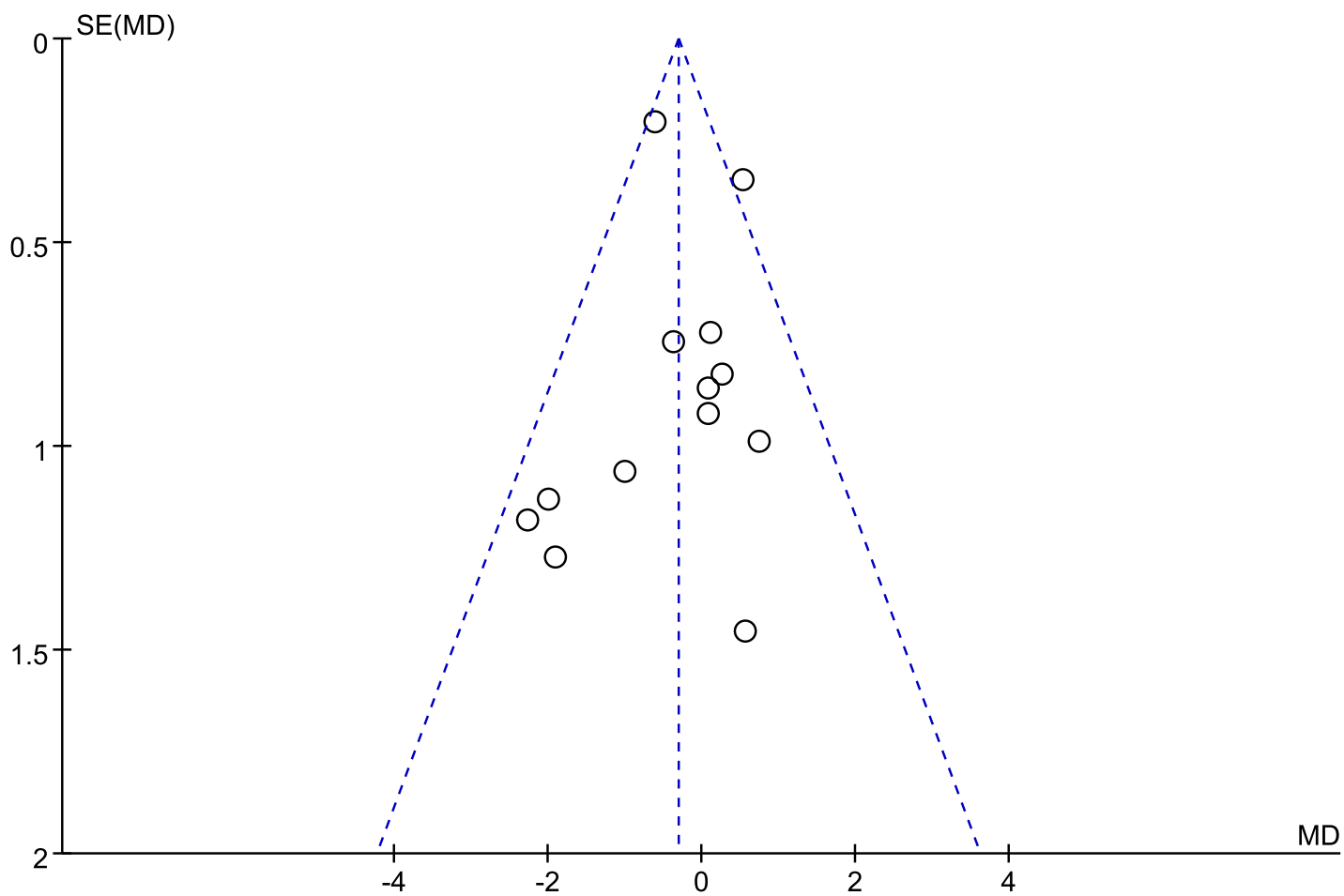

Supplement: Multimedia Appendix 12 [file mhealth_v12i1e51478_app12.pdf]

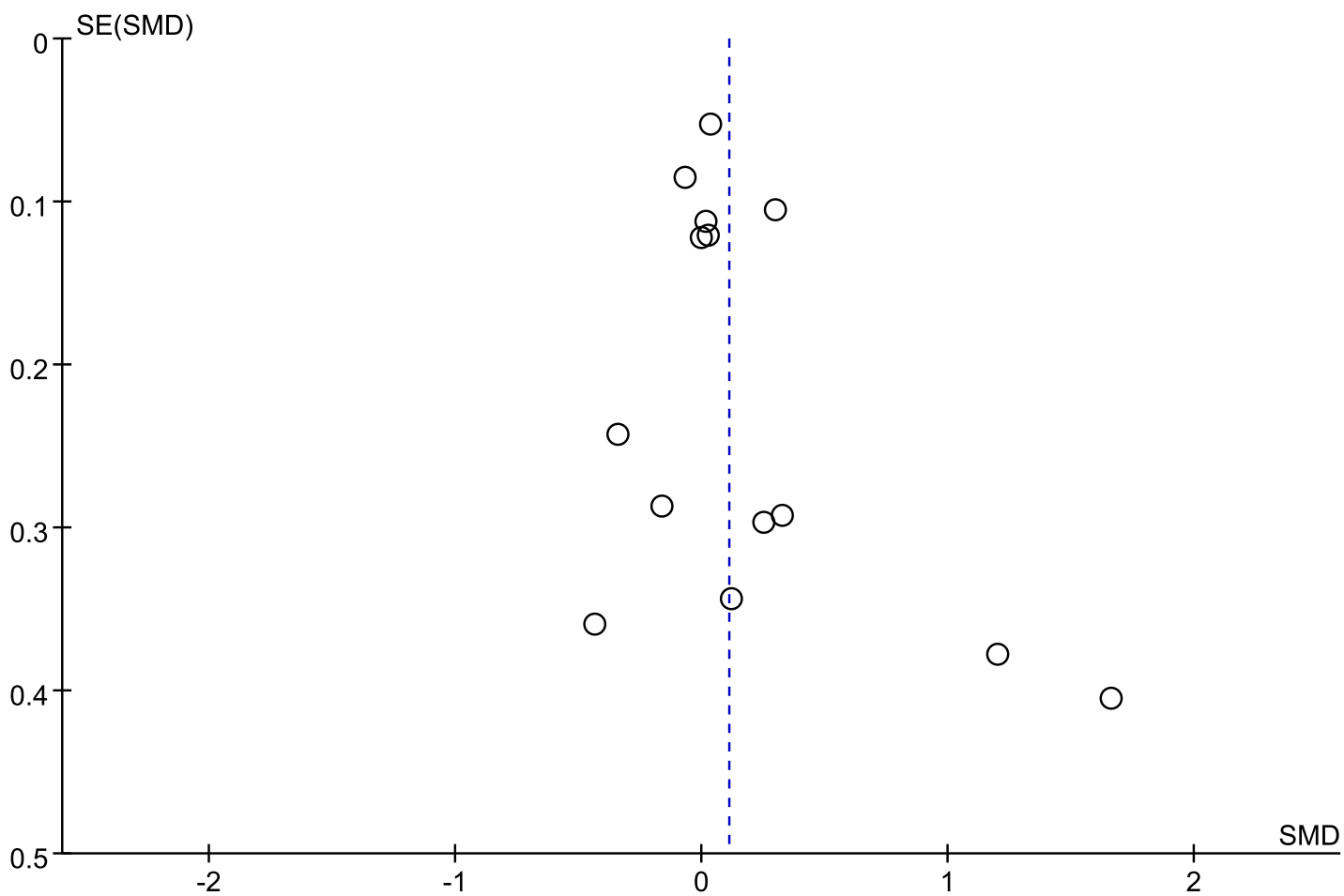

Supplement: Multimedia Appendix 13 [file mhealth_v12i1e51478_app13.pdf]

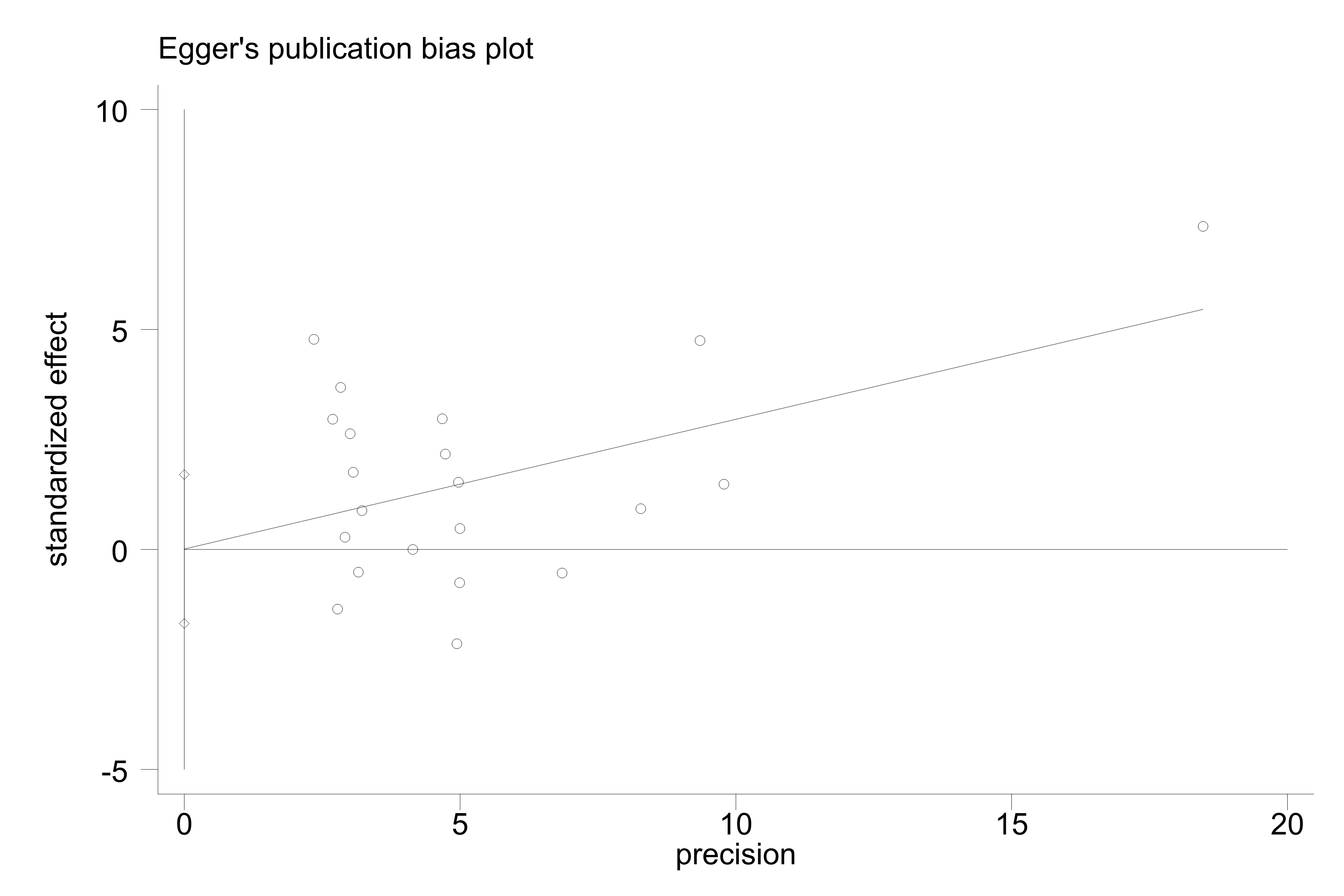

Supplement: Multimedia Appendix 14 [file mhealth_v12i1e51478_app14.png]

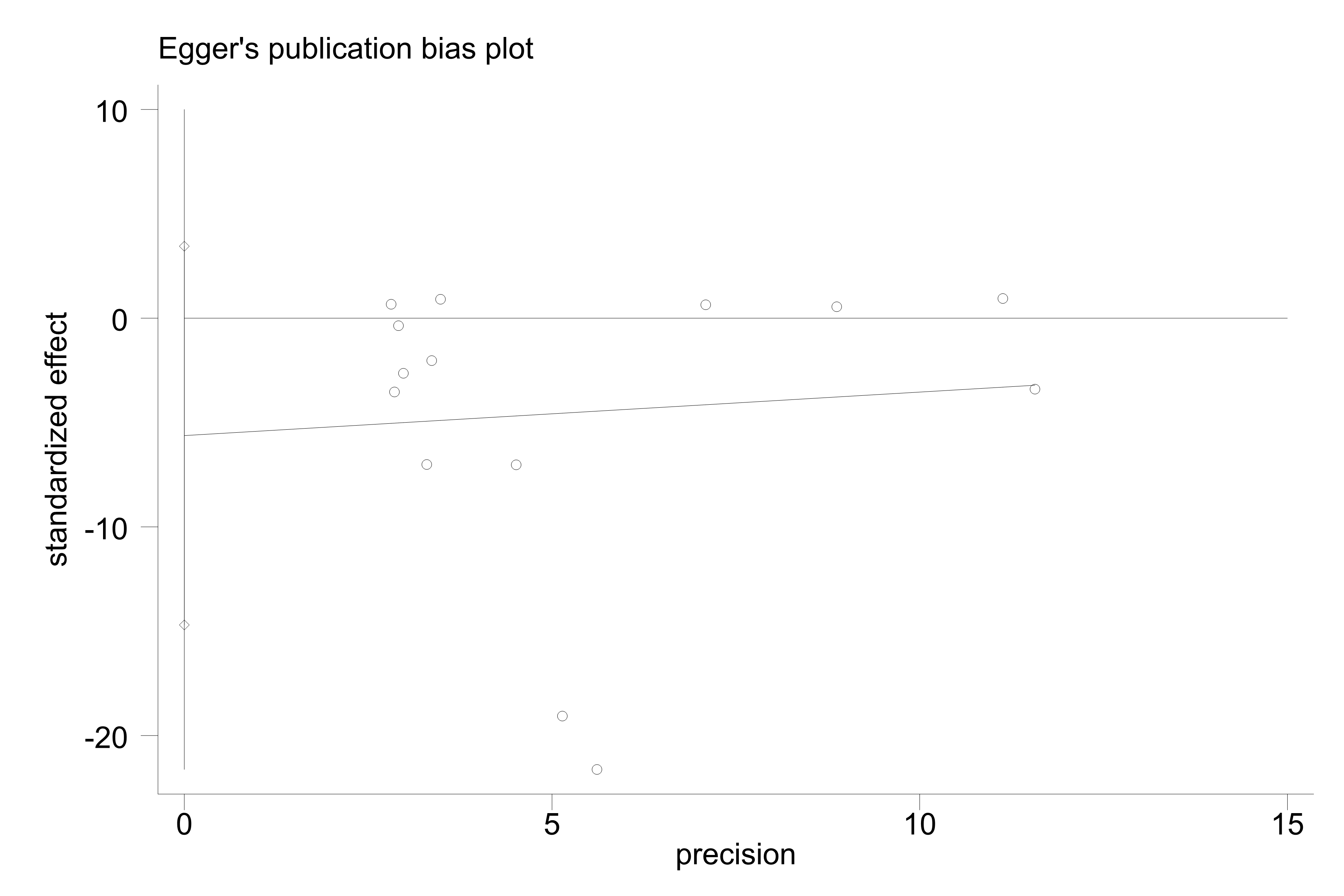

Supplement: Multimedia Appendix 15 [file mhealth_v12i1e51478_app15.png]

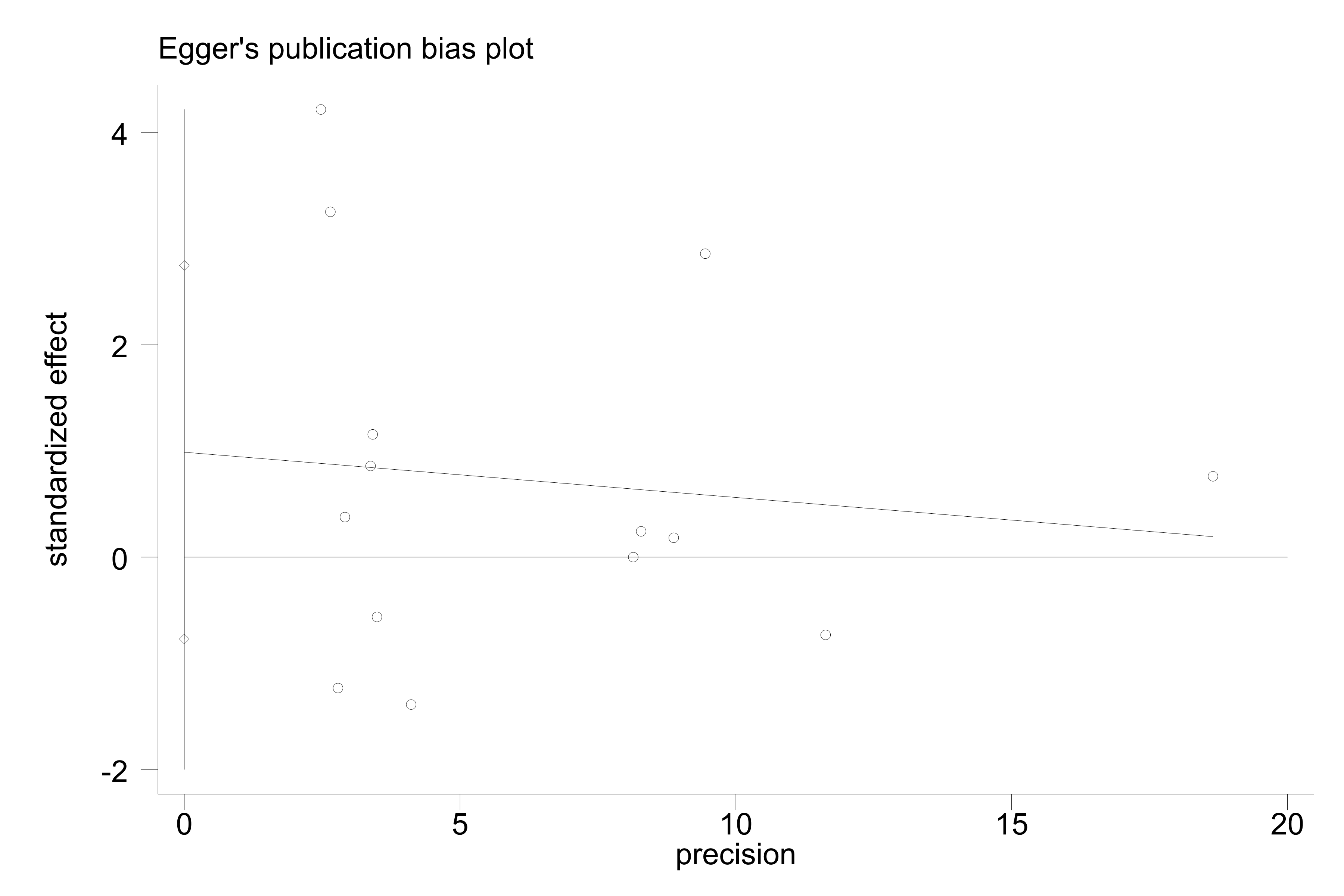

Supplement: Multimedia Appendix 16 [file mhealth_v12i1e51478_app16.png]

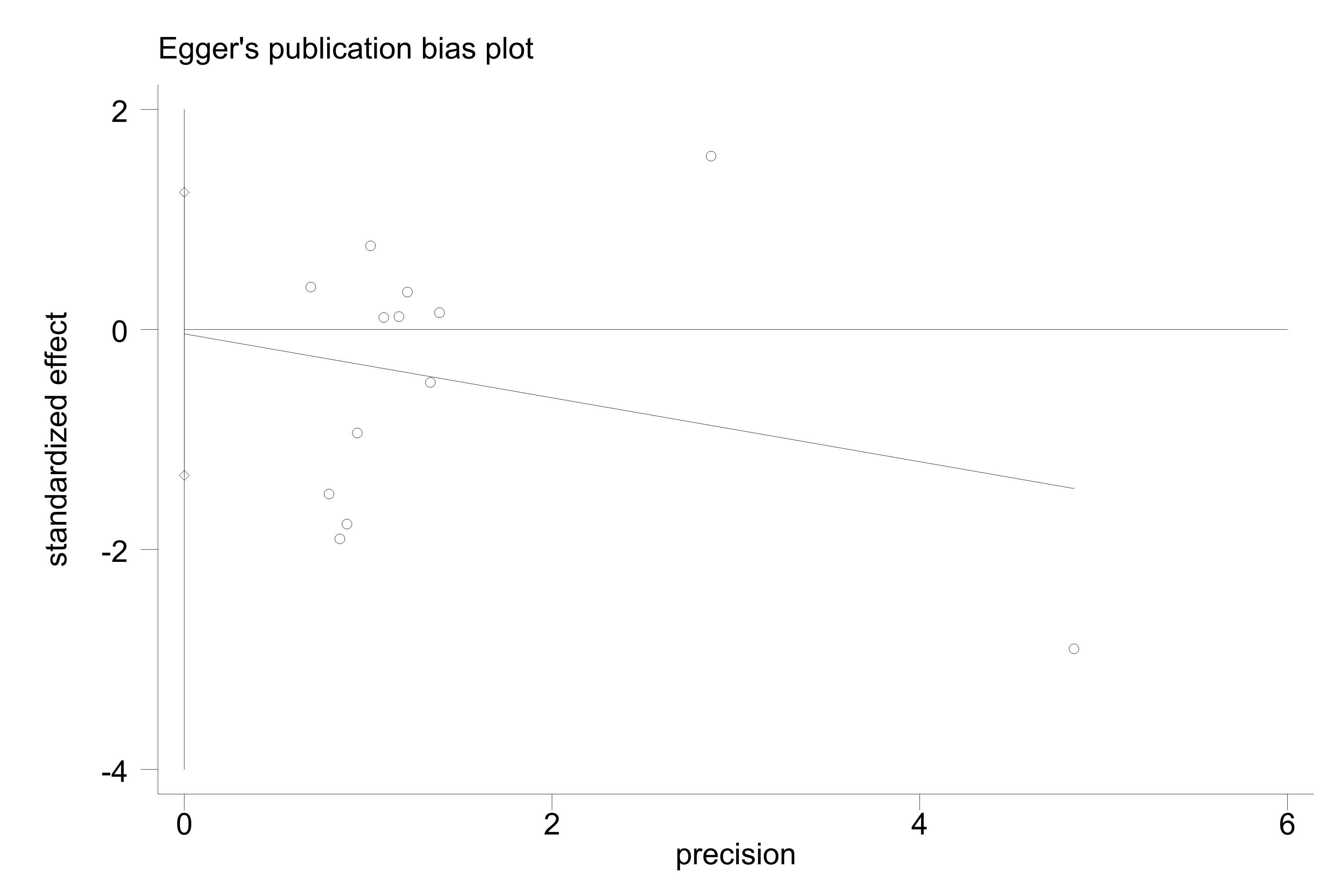

Supplement: Multimedia Appendix 17 [file mhealth_v12i1e51478_app17.png]
