# Supplementary material for: Effectiveness of mHealth App–Based Interventions for Increasing Physical Activity and Improving Physical Fitness in Children and Adolescents: Systematic Review and Meta-Analysis
Source: JMIR Mhealth Uhealth. 2024 Apr 30;12:e51478. doi: 10.2196/51478 (PMC11094610; doi:10.2196/51478)
Supplement: Multimedia Appendix 6 [file mhealth_v12i1e51478_app6.pdf]

**Table 2.** Summary of subgroup analysis results of mHealth app-based interventions on TPA.

| Potential modifiers | Studies, | Tests of heterogeneity | | | Results of the Meta-analysis | |
|---|---|---|---|---|---|---|
| | n | Q | P | $I^2$(%) | Effect size（95%CI） | P-value |
| TPA | | | | | | |
| Pooled effect size | 21 | 79.93 | <0.00001 | 75 | 0.29（0.13，0.45） | 0.0004 |
| Age(years) | | | | | | |
| 3～6 | 2 | 0.16 | 0.69 | 0 | -0.06（-0.44， 0.32） | 0.76 |
| 7～12 | 4 | 2.79 | 0.43 | 0 | 0.42（0.33，0.52） | <0.00001 |
| 13～18 | 15 | 60.06 | <0.00001 | 77 | 0.29（0.07，0.51） | 0.01 |
| Types of apps | | | | | | |
| Research apps | 9 | 29.61 | 0.0002 | 73 | 0.13（-0.12，0.38） | 0.30 |
| Commercial apps | 10 | 40.68 | <0.00001 | 78 | 0.51（0.21, 0.82） | 0.0009 |
| Types of intervention | | | | | | |
| stand-alone apps | 16 | 54.48 | <0.00001 | 72 | 0.23（0.01, 0.44） | 0.04 |
| concerted intervention | 5 | 13.03 | 0.01 | 69 | 0.44（0.22, 0.65） | <0.00001 |
| Theoretical paradigm | | | | | | |
| SCT | 3 | 9.02 | 0.01 | 78 | 0.58（-0.15, 1.31） | 0.12 |
| combination of SCT and other theories | 3 | 12.09 | 0.002 | 83 | 0.19（-0.15, 0.53） | 0.28 |
| SDT | 2 | 2.59 | 0.11 | 61 | 1.51（0.62, 2.40） | 0.0009 |
| combination of SDT and other theories | 2 | 1.67 | 0.20 | 40 | 0.55（-0.03, 1.13） | 0.06 |
| SRT | 5 | 8.97 | 0.11 | 44 | -0.07（-0.32, 0.18） | 0.58 |
| The number of BCT clusters | | | | | | |
| 1～3 | 6 | 30.23 | <0.0001 | 83 | 0.57（-0.02, 1.15） | 0.06 |
| 4 | 8 | 25.23 | 0.0007 | 72 | 0.07（-0.17, 0.32） | 0.57 |
| 7～10 | 3 | 10.21 | 0.006 | 80 | 0.60（-0.08, 1.26） | 0.006 |
| Intervention duration | | | | | | |
| 2～4 | 4 | 10.86 | 0.01 | 72 | 1.01（0.34, 1.67） | 0.003 |
| 8～12 | 9 | 20.97 | 0.007 | 62 | 0.23（0.02, 0.43） | 0.03 |
| 20～48 | 7 | 26.02 | 0.0002 | 77 | 0.20（-0.04, 0.43） | 0.10 |
