# Supplementary material for: Effectiveness of mHealth App–Based Interventions for Increasing Physical Activity and Improving Physical Fitness in Children and Adolescents: Systematic Review and Meta-Analysis
Source: JMIR Mhealth Uhealth. 2024 Apr 30;12:e51478. doi: 10.2196/51478 (PMC11094610; doi:10.2196/51478)
Supplement: Multimedia Appendix 7 [file mhealth_v12i1e51478_app7.pdf]

Table 3. Summary of subgroup analysis results of mHealth app-based interventions on SB.

Potential modifiers	Studies, n	Tests of heterogeneity			Results of the Meta-analysis	
		Q	P	I^2(%)	Effect size（95%CI）	P-value
SB						
Pooled effect size	14	776.42	<0.00001	98	-0.97（-1.67, -0.28）	0.006
Age(years)						
3～6	4	76.11	<0.00001	96	-0.92（-1.65, -0.19）	0.01
7～12	2	0.31	0.58	0	-3.78（-4.04, -3.52）	<0.00001
13～18	8	25.19	0.0007	72	-0.21（-0.51, 0.10）	0.18
Types of apps						
Research apps	8	689.19	<0.00001	99	-1.38（-2.46, -0.30）	0.01
Commercial apps	5	19.86	0.0005	80	-0.35（-0.88, 0.18）	0.20
Types of interv-ention						
stand-alone apps	7	94.94	<0.00001	94	-0.45（-0.96, 0.05）	0.08
concerted intervention	7	525.73	<0.00001	99	-1.47（-2.88, -0.06）	0.04
Theoretical paradigm						
SCT	6	58.86	<0.00001	92	-0.64（-1.16, -0.13）	0.01
combination of SCT and other theories	2	402.87	<0.00001	100	-2.48（-5.21, 0.25）	0.07
SRT	2	0.51	0.48	0	0.05（-0.44, 0.53）	0.84
The number of BCT clusters						
1～3	5	45.12	<0.00001	91	-0.41（-111, 0.30）	0.26
4	7	710.54	<0.00001	99	-1.36（-2.52, -0.19）	0.02
7～10	2	0.50	0.48	0	-1.03（-1.51, -0.56）	<0.0001
Intervention duration						
8～12	8	84.18	<0.00001	92	-0.63 (-1.26, 0.01)	0.05
20～48	6	690.35	<0.00001	99	-1.42 (-2.62, -0.21)	<0.00001
