# Supplementary material for: Effectiveness of mHealth App–Based Interventions for Increasing Physical Activity and Improving Physical Fitness in Children and Adolescents: Systematic Review and Meta-Analysis
Source: JMIR Mhealth Uhealth. 2024 Apr 30;12:e51478. doi: 10.2196/51478 (PMC11094610; doi:10.2196/51478)
Supplement: Multimedia Appendix 8 [file mhealth_v12i1e51478_app8.pdf]

Table 4. Summary of subgroup analysis results of mHealth app-based interventions on MVPA.

Potential modifiers	Studies,	Tests of heterogeneity			Results of the Meta-analysis	
	n	Q	P	I^2(%)	Effect size（95%CI）	P-value
SB						
Pooled effect size	14	39.06	0.0002	67	0.11（-0.04, 0.25）	0.14
Age(years)						
3～6	3	1.77	0.41	0	-0.05（-0.18, 0.07）	0.41
7～12	3	5.35	0.07	63	0.11（-0.06, 0.28）	0.21
13～18	7	23.95	0.0005	75	0.42（0.01, 0.83）	0.04
Types of apps						
Research apps	6	10.50	0.06	52	0.02（-0.14, 0.17）	0.84
Commercial apps	8	25.28	0.0001	80	0.43（-0.09, 0.95）	0.11
Types of interv-ention						
stand-alone apps	7	29.56	<0.00001	80	0.30（-0.15, 0.75）	0.19
concerted intervention	7	9.08	0.17	34	0.06（-0.05, 0.16）	0.28
Theoretical paradigm						
SCT	4	1.90	0.59	0	-0.06（-0.19, 0.07）	0.35
combination of SCT and other theories	3	4.45	0.11	55	0.12（-0.08, 0.31）	0.24
SDT	3	7.96	0.02	75	1.03（0.22, 1.83）	0.01
SRT	2	1.26	0.26	21	-0.14（-0.63, 0.35）	0.61
The number of BCT clusters						
1～3	8	27.21	0.0003	74	0.24（-0.04，0.53）	0.09
4	5	10.49	0.03	62	0.01（-0.20,　0.21）	0.95
Intervention duration						
2～4	2	0.70	0.40	0	1.42（0.88, 1.96）	<0.00001
8～12	7	5.83	0.44	0	-0.01 (-0.18, 0.16）	0.92
20～48	5	7.65	0.11	48	0.05 (-0.06,　0.16）	0.34
