# Supplementary material for: Effectiveness of mHealth App–Based Interventions for Increasing Physical Activity and Improving Physical Fitness in Children and Adolescents: Systematic Review and Meta-Analysis
Source: JMIR Mhealth Uhealth. 2024 Apr 30;12:e51478. doi: 10.2196/51478 (PMC11094610; doi:10.2196/51478)
Supplement: Multimedia Appendix 9 [file mhealth_v12i1e51478_app9.pdf]

Table 5. Summary of subgroup analysis results of mHealth app-based interventions on BMI.

Potential modifiers	Studies, n	Tests of heterogeneity			Results of the Meta-analysis	
		Q	P	I^2(%)	Effect size（95%CI）	P-value
BMI						
Pooled effect size	13	17.75	0.12	32	-0.31（-0.60, -0.01）	0.04
Age(years)						
7～12	5	2.92	0.57	0	-0.59（-0.96，-0.22）	0.002
13～18	8	8.98	0.25	22	0.03（-0.60，0.65）	0.93
Types of apps						
Research apps	6	1.86	0.87	0	-0.04（-0.79，0.72）	0.93
Commercial apps	5	8.06	0.09	50	-0.21（-1.17，0.75）	0.66
Types of intervention						
stand-alone apps	4	1.35	0.72	0	0.34（-0.20，0.87）	0.22
concerted intervention	9	8.42	0.39	5	-0.59（-1.03，-0.15）	0.009
Theoretical paradigm						
SCT	3	2.49	0.29	20	-1.36（-2.99，0.27）	0.10
combination of SCT and other theories	3	0.79	0.67	0	-0.18（-1.24，0.87）	0.73
The number of BCT clusters						
1～4	6	1.82	0.87	0	-0.04（-0.79，0.72）	0.86
7	3	8.03	0.02	75	-0.94（-3.04，1.15）	0.38
Intervention duration						
8～12	5	3.60	0.46	0	0.33（-0.22，0.88）	0.24
20～48	7	6.73	0.35	11	-0.57（-1.09, -0.05）	0.03
